# Structural Optimisation of an Amphibian BBI-Type Peptide Enhances Endothelial Protection Against Methylglyoxal-Induced Injury Through Coordinated Regulation of Glyoxalase-Mediated Detoxification and Redox Homeostasis

**DOI:** 10.3390/biom16081157

**Published:** 2026-08-09

**Authors:** Ying Wang, Wenyu Wu, Wudi Wang, Weichang Li, Zhenggang Yue, Chengbang Ma, Lei Wang, Mei Zhou, James F. Burrows, Tianbao Chen, Fanxing Xu

**Affiliations:** 1 Shaanxi Province Key Laboratory of New Drugs and Chinese Medicine Foundation Research, School of Pharmacy, Shaanxi University of Chinese Medicine, Xi’an 712046, China; wangying011@sntcm.edu.cn (Y.W.); 1501019@sntcm.edu.cn (Z.Y.); 2 Wuya College of Innovation, Shenyang Pharmaceutical University, Shenyang 110016, China; 202383093@stu.syphu.edu.cn; 3 Natural Drug Discovery Group, School of Pharmacy, Queen’s University Belfast, Belfast BT9 7BL, UK; wwang35@qub.ac.uk (W.W.); wli25@qub.ac.uk (W.L.); c.ma@qub.ac.uk (C.M.); l.wang@qub.ac.uk (L.W.); m.zhou@qub.ac.uk (M.Z.); j.burrows@qub.ac.uk (J.F.B.)

**Keywords:** Bowman–Birk inhibitor peptide, methylglyoxal, oxidative stress, endothelial dysfunction, PI3K/AKT/GSK3β/Nrf2 signalling

## Abstract

Impaired endothelial function, excessive oxidative stress, and persistent bacterial infection collectively contribute to delayed healing of diabetic chronic wounds. Methylglyoxal (MGO)-induced metabolic stress is a critical driver of endothelial injury; however, effective multifunctional strategies capable of restoring vascular function and maintaining cellular homeostasis remain limited. In this study, the endothelial protective potential of an amphibian-derived Bowman–Birk inhibitor (BBI)-type peptide, OSTI-1872, and its rationally designed structural analogues were investigated using an MGO-induced injury model in human umbilical vein endothelial cells (HUVECs). Among the tested peptides, the optimised analogue OSTI-2337 exhibited superior protective activity. OSTI-2337 markedly attenuated MGO-induced oxidative stress, restored nitric oxide bioavailability, enhanced VEGF expression, promoted endothelial migration and tube formation, and reduced oxidative DNA damage. Mechanistically, these effects were associated with coordinated regulation of MGO detoxification and redox homeostasis, as evidenced by enhanced GLO1 expression and modulation of the PI3K/AKT/GSK3β/Nrf2 axis, accompanied by increased expression of downstream antioxidant proteins HO-1 and NQO1. In addition, OSTI-1872 and OSTI-2337 displayed antibacterial activity against representative bacterial strains, suggesting their potential advantages for complex diabetic wound environments. Collectively, these findings demonstrate that structural optimisation significantly enhances the biological activity of amphibian BBI-type peptides and identify OSTI-2337 as a multifunctional peptide candidate capable of integrating endothelial protection, MGO detoxification, redox regulation, and antibacterial activity for the management of diabetes-associated vascular injury and chronic wound complications.

## 1. Introduction

Diabetic vascular complications and chronic wounds represent major clinical challenges associated with diabetes, in which endothelial dysfunction serves as a critical pathological event [1]. Impaired endothelial function disrupts nitric oxide availability, angiogenic capacity, and vascular repair, thereby contributing to poor tissue regeneration and delayed wound healing. In addition, persistent bacterial colonisation further aggravates inflammatory responses and oxidative damage within diabetic wounds [2,3,4]. Therefore, therapeutic strategies capable of simultaneously improving endothelial function and controlling pathological stress responses are highly desirable.

Methylglyoxal (MGO), a highly reactive dicarbonyl metabolite generated during glucose metabolism, is increasingly recognised as an important mediator of diabetic vascular injury. Excessive MGO accumulation promotes endothelial dysfunction through oxidative stress induction, protein glycation, and disruption of cellular homeostasis. Under physiological conditions, MGO is primarily eliminated by the glyoxalase system, in which glyoxalase 1 (GLO1) functions as the rate-limiting detoxification enzyme. However, impaired GLO1 activity under diabetic conditions facilitates MGO accumulation and amplifies oxidative injury [5]. These findings indicate that effective intervention against MGO-associated endothelial damage may require coordinated regulation of MGO detoxification and antioxidant defence mechanisms rather than targeting oxidative stress alone.

Natural peptides derived from amphibian skin secretions have attracted increasing attention as promising bioactive molecules due to their structural diversity and multifunctional biological activities, including antimicrobial, anti-inflammatory, antioxidant, and immunomodulatory effects [6]. Unlike conventional small-molecule antioxidants, bioactive peptides can be structurally engineered to optimise activity, selectivity, and biological stability [7]. Although amphibian-derived peptides have been extensively investigated in antimicrobial and anticancer fields, their potential roles in metabolic stress-associated vascular disorders remain largely unexplored [8,9,10,11,12].

Bowman–Birk inhibitors (BBIs) are cysteine-rich serine protease inhibitors originally identified in plants, while structurally related BBI-type peptides have also been discovered in amphibian secretions [13]. These peptides possess highly conserved disulfide-stabilised structures and have demonstrated diverse biological activities beyond protease inhibition, including regulation of inflammation and oxidative responses [14,15,16,17]. However, whether amphibian BBI-type peptides can protect endothelial cells against MGO-induced metabolic injury remains unknown. Furthermore, the feasibility of enhancing their vascular protective activity through rational structural optimisation and the underlying molecular mechanisms have not been established.

OSTI-1872 was previously identified as an amphibian-derived Bowman–Birk inhibitor-type peptide and reported for its antibacterial activity [18]. However, its potential protective effect against methylglyoxal (MGO)-induced endothelial dysfunction has not been investigated. In the present study, OSTI-1872 was used as the parent peptide for structural optimisation, and three newly designed analogues were synthesised and systematically evaluated for their endothelial protective effects. Using an MGO-induced HUVEC injury model, we demonstrated that the optimised analogue OSTI-2337 improved endothelial function through coordinated regulation of oxidative defence and MGO detoxification pathways. The antibacterial activity of these peptides was also evaluated to explore their potential relevance to diabetic wound-associated complications. This study provides new insights into the therapeutic potential of amphibian BBI-type peptides and highlights structural optimisation as an effective strategy for developing multifunctional peptide candidates against diabetes-related vascular injury.

## 2. Materials and Methods

### 2.1. Peptide Design and Solid-Phase Synthesis

To optimise the physicochemical properties and biological activity of the parent peptide while preserving the characteristic BBI-type scaffold, a series of analogues was designed through targeted amino acid substitution, motif replacement, and sequence extension. All peptides were synthesised by automated standard Fmoc-based solid-phase peptide synthesis (SPPS) using a Tribute^®^ peptide synthesiser (Protein Technologies, Tucson, AZ, USA).

Peptides were performed on resins with a loading of ~0.3 mmol/g: MBHA resin for peptides with C-terminal amidation, or Wang resin for peptides with a free C-terminal carboxyl group. Amino acids were sequentially coupled, with 2-(1H-benzotriazole-1-yl)-1,1,3,3-tetramethyluronium hexafluorophosphate (HBTU) as the coupling agent, mixed with amino acids at a ratio of 0.3 mmol × 2.5 equivalences. After synthesis, the peptides were cleaved from the resin. The cleavage mixture consisted of 94% trifluoroacetic acid (TFA), 2% double-distilled water (ddH_2_O), 2% triisopropylsilane (TIS), and 2% 1,2-ethylenedithiol (EDT). The cleavage products were precipitated with cold diethyl ether, collected by centrifugation, dried, and finally lyophilised and stored at −20 °C for later use.

### 2.2. Physicochemical Properties and Secondary Structure Prediction

The physicochemical properties of the peptides were calculated using the online BACHEM Peptide Calculator (https://www.bachem.com/knowledge-center/peptide-calculator/ (accessed on 10 April 2024)). The theoretical net charge values were calculated at pH 7.0. The secondary structure of the peptides was predicted using the online web server PEP-FOLD 3.5 (https://mobyle.rpbs.univ-paris-diderot.fr/cgi-bin/portal.py#forms::PEP-FOLD3 (accessed on 10 April 2024)).

### 2.3. Peptide Purification and Identification

The crude peptides were purified by reversed-phase high-performance liquid chromatography (RP-HPLC) using a LUNA C-5 preparative column (250 mm × 10 mm, Phenomenex, Torrance, CA, USA). Mobile phase A was deionised water containing 0.5% TFA, and mobile phase B was a mixture of 80% acetonitrile, 19.5% deionised water, and 0.5% TFA. Gradient elution separation was performed based on the differences in hydrophobicity of the peptides.

The purified peptides were analysed and identified using matrix-assisted laser desorption/ionisation time-of-flight mass spectrometry (MALDI-TOF MS) (Voyager DE, Perseptive BioSystems, Framingham, MA, USA). The matrix solution was α-cyano-4-hydroxycinnamic acid (CHCA) solvent (10 mg/mL) containing 70% acetonitrile, 30% water, and 0.1% TFA. In total, 2 μL of HPLC fraction and 1 μL of CHCA solution were loaded onto a plate, and the sample was loaded after the droplets dried. The mass-to-charge ratio (*m*/*z*) was recorded to identify the target peptide.

### 2.4. Peptide Secondary Structure Analysis

The secondary structure of the peptides was analysed by circular dichroism (CD) spectroscopy using a J-815 spectrometer (Jasco, Tokyo, Japan). The peptides were dissolved in 20 mM ammonium acetate (pH 7.0) to prepare a 100 μM stock solution, which was then diluted with deionised water and trifluoroethanol (TFE) to generate peptide solutions under the aqueous phase and membrane-mimicking hydrophobic conditions (pH 6.8), respectively. The final peptide concentration used for CD measurements was 50 μM. CD spectra were scanned in the range of 190–260 nm, with a quartz cuvette path length of 1 mm, a scan speed of 200 nm/min, a bandwidth of 1 nm, and a data interval of 0.5 nm. The α-helix and β-sheet ratios were analysed by characteristic peaks.

### 2.5. In Vitro Antibacterial Test

The antibacterial activity of peptides was determined using two indicators: minimum inhibitory concentration (MIC) and minimum bactericidal concentration (MBC). MIC is defined as the lowest peptide concentration that can inhibit the visible growth of bacteria; MBC refers to the lowest peptide concentration that can kill all bacteria in the test system. Eight bacterial strains were used in this experiment, including *Escherichia coli* (ATCC CRM8739), *Pseudomonas aeruginosa* (ATCC CRM9027), *Klebsiella pneumoniae* (ATCC CRM43861), *Acinetobacter baumannii* (BAA747), *Staphylococcus aureus* (ATCC CRM6538), *Enterococcus faecalis* (NCTC 12697), methicillin-resistant *Staphylococcus aureus* (MRSA, NCTC 12493), and *Candida albicans* (ATCC 10231).

The strains were removed from the −20 °C freezer and inoculated into Erlenmeyer flasks containing culture medium. They were then incubated on a shaker at 37 °C and 120 rpm for 16–20 h (for genuine strains, incubation was at 26 °C). Subsequently, 0.5 mL of the above bacterial culture was transferred to a McCartney bottle containing 20 mL of broth medium and cultured a second time under the same conditions. The second-cultured bacterial culture was diluted 200-fold with fresh medium, and the concentration was adjusted to a working concentration of 5 × 10^5^ CFU/mL based on the absorbance (OD value) at 550 nm. The target peptide was dissolved in DMSO to prepare a concentration gradient, which was used to test the antibacterial activity of the diluted bacterial culture. Norfloxacin (20 μg/mL, 62.6 μM) or amphotericin B (10 μg/mL, 10.8 μM) were set as positive controls, and negative controls and solvent controls were also set. The culture media of each experimental group were dispensed into 96-well plates and incubated at incubation for 20–24 h. The absorbance value of each well at 550 nm was measured using a Synergy HT plate reader (Bio-Tek, Minneapolis, MN, USA).

### 2.6. Cell Culture

Human umbilical vein endothelial cells (HUVECs) were purchased from CHI Scientific Co., Ltd. (Jiangyin, Jiangsu, China; Cat. No. 7-1074) and cultured in RPMI-1640 medium supplemented with 10% foetal bovine serum (FBS), 100 U/mL penicillin, and 100 μg/mL streptomycin. Cells were maintained at 37 °C in a humidified incubator containing 5% CO_2_ and passaged using trypsin digestion upon reaching 80–90% confluence.

### 2.7. NO Content Determination

The nitric oxide (NO) content in cell culture supernatant was determined using the Griess reagent method. The experiment was performed according to the kit instructions (Beyotime Biotechnology, Shanghai, China). A summary of the steps is as follows: Griess Reagent I and Griess Reagent II were equilibrated to room temperature, and NO standards were serially diluted (1–100 μM) with the same solution as the samples to plot a standard curve. After HUVECs were cultured in 96-well plates and treated with the appropriate drugs, 50 μL of culture supernatant from each well was collected and added to a new 96-well plate, along with the same volume of standard solution. Subsequently, 50 μL of Griess Reagent I and 50 μL of Griess Reagent II were added sequentially, mixed thoroughly, and reacted at room temperature. The absorbance (OD) was measured at 540 nm using a multi-mode microplate reader. The NO concentration in the samples was calculated based on the standard curve.

### 2.8. Immunofluorescence Confocal Microscopy

Immunofluorescence confocal microscopy was performed to evaluate protein expression and localisation based on antigen–antibody specific binding. HUVECs were seeded onto sterilised glass coverslips placed in 24-well plates at a density of 1 × 10^5^ cells per well and cultured for 48 h. Cells were then treated with the indicated conditions for 24 h. After treatment, cells were washed three times with PBS and fixed with 4% paraformaldehyde at room temperature for 30 min. Following fixation, cells were permeabilised with 0.15% Triton X-100 for 15 min and then blocked with 10% goat serum for 30 min at room temperature. Subsequently, cells were incubated overnight at 4 °C with primary antibodies against VEGF (dilution 1:200) (Beyotime Biotechnology, Shanghai, China). After washing with PBST, cells were incubated with a fluorophore-conjugated secondary antibody (Alexa Fluor 488-labelled goat anti-rabbit IgG, 1:500) (Proteintech Group, Rosemont, IL, USA) for 2.5 h at room temperature in the dark. The green fluorescence signal represents VEGF protein expression. Cell nuclei were counterstained with DAPI (1:8000) for 10 min. After washing, coverslips were mounted using antifade mounting medium and sealed. Images were acquired using a laser confocal microscope under identical acquisition settings for all groups. Quantitative analysis of fluorescence intensity was performed using ImageJ software (Fiji/ImageJ, version 1.54).

### 2.9. Tube Formation Assay

The Matrigel was thawed on ice and mixed with a pre-chilled pipette tip. 20–30 μL was added to each well of a 24-well plate. The plate was then sealed and incubated overnight at 4 °C. The next day, the plate was transferred to a 37 °C, 5% CO_2_ incubator and incubated for 30 min until the Matrigel was completely solidified.

HUVECs were treated with 600 μM MGO in the presence or absence of 0.1 μM OSTI-1872 or OSTI-2337 for 24 h. Cells were then digested with trypsin and centrifuged at 1000 rpm for 5 min to collect the cells. The supernatant was discarded, and the cells were resuspended in RPMI-1640 complete medium and counted. Cells were then seeded at a density of 1.5 × 10^5^ cells/well on solidified Matrigel, with three replicates per group. The plates were incubated at 37 °C, 5% CO_2_ for 12 h, and the vascular-like structures were observed and photographed under an optical microscope. ImageJ software (Fiji/ImageJ, version 1.54) was used to analyse images and quantitatively calculate the total tube length, number of branch points, number of junctions, and number of meshes to evaluate the in vitro angiogenesis capacity of the cells.

### 2.10. Wound Healing Assay

HUVECs in logarithmic growth phase were trypsinised and seeded at a density of 4 × 10^5^ cells/mL in 6-well culture plates and cultured at 37 °C in a 5% CO_2_ incubator until the cell monolayers reached confluence. Then, a 200 μL sterile pipette tip was used to vertically draw straight lines on the cell monolayer to form cell scratches. The cells were gently washed three times with PBS to remove detached cells from the scratched areas. Serum-free medium containing the appropriate drug was then added for further culture. Images of the scratches were taken at the same field of view at 0 h, 24 h, and 48 h. ImageJ software (Fiji/ImageJ, version 1.54) was used to quantitatively analyse the scratch area and calculate cell migration rate. The formula for calculating cell migration rate is as follows:

Migration rate (%) = (A_0h_ − A_t_)/(A_0h_) × 100%

where A_0h_ represents the scratch area at 0 h, and A_t_ corresponds to the scratch area at the time point (24 h or 48 h).

### 2.11. Intracellular ROS Level Detection

#### 2.11.1. Fluorescence Microscopy Detection of ROS

Intracellular reactive oxygen species (ROS) levels were detected using the 2,7-dichlorodihydrofluorescein diacetate (DCFH-DA) fluorescent probe. DCFH-DA can freely cross the cell membrane and is hydrolysed by esterases within the cell to generate non-fluorescent DCFH, which is then oxidised to green, fluorescent DCF under the action of ROS. Its fluorescence intensity is positively correlated with intracellular ROS levels.

HUVECs were seeded at 3 × 10^5^ cells/well in 6-well culture plates containing sterile coverslips. After treatment with the appropriate drugs, the culture medium was discarded, and the cells were washed three times with PBS. Then, the DCFH-DA fluorescent probe was added according to the kit instructions, and the cells were incubated at 37 °C in the dark for 1 h. After incubation, the cells were washed three times with PBS to remove any probes that had not entered the cells.

The nuclei were then stained with DAPI staining solution (1:2000) for 10 min, followed by washing three times with PBS. Anti-fluorescence quenching mounting medium was added to a glass slide, and the coverslip was inverted with the cell side down and sealed with nail polish. The samples were observed using a laser confocal microscope, and fluorescence images were acquired. Intracellular ROS levels were assessed by analysing the DCF fluorescence intensity.

#### 2.11.2. Flow Cytometry Detection of ROS

To further quantify intracellular reactive oxygen species (ROS) levels, flow cytometry combined with the DCFH-DA fluorescent probe was used for detection.

HUVECs were seeded at a density of 3 × 10^5^ cells/well in 6-well culture plates and collected after treatment with the appropriate drugs. After discarding the culture medium, trypsin was added to digest the cells. The cell suspension was then transferred to a 15 mL centrifuge tube and centrifuged at 1500 rpm for 5 min to collect the cell pellet. The pellet was washed twice with PBS (pH 7.4). The DCFH-DA fluorescent probe was then prepared as a working solution with PBS and added to the centrifuge tube to resuspend the cell pellet. The pellet was incubated at 37 °C and 5% CO_2_ in the dark for 45 min. After incubation, the cells were washed 2–3 times with PBS to remove unbound probe. Finally, the cells were resuspended in 1 mL PBS and analysed using flow cytometry (Becton, Dickinson and Company, Franklin Lakes, NJ, USA). Intracellular ROS levels were quantitatively assessed by analysing the DCF fluorescence intensity.

### 2.12. Antioxidant Indicators Detection (T-SOD and MDA)

Antioxidant indicators were evaluated by measuring total superoxide dismutase (T-SOD) activity and malondialdehyde (MDA) content using commercial assay kits (Wanleibio, Shenyang, China) according to the manufacturer’s instructions. T-SOD activity was determined using the xanthine oxidase method. In this assay, xanthine is catalysed by xanthine oxidase to generate superoxide anion radicals (O_2_^−^·), which subsequently react with hydroxylamine to produce nitrite. The nitrite then forms a coloured complex in the presence of a chromogenic agent, and the absorbance was measured at 550 nm using a microplate reader. T-SOD activity is inversely proportional to the formation of the coloured product. MDA content was measured using the thiobarbituric acid (TBA) method. Under acidic and high-temperature conditions, MDA reacts with TBA to form a thiobarbituric acid reactive substance (TBARS), resulting in a reddish-brown complex. The absorbance was measured at 532 nm to reflect the level of lipid peroxidation.

### 2.13. Western Blot Analysis

To further investigate the effect of peptides on the molecular mechanisms related to MGO-induced vascular endothelial cell injury, Western blot analysis was performed to detect the expression levels of proteins in related signalling pathways using a chemiluminescence detection system (Bio-Rad Laboratories, Hercules, CA, USA), and band intensities were quantified using Image Lab software (version 6.1, Bio-Rad, Hercules, CA, USA).

#### 2.13.1. Protein Extraction

Cells in logarithmic growth phase were seeded at a density of 3 × 10^5^ cells/well in 6-well culture plates and cultured for 24 h. Subsequently, cells were treated with the corresponding drugs according to the experimental groups for 24 h. After treatment, cells were collected into 1.5 mL EP tubes. In total, 100 μL of RIPA lysis buffer (containing 1% protease inhibitor and phosphatase inhibitor) was added to each tube for lysis. Lysis was performed under ice bath conditions for 40 min, with vortexing for 15 s every 10 min. After lysis, the cells were centrifuged at 12,000× *g* for 10 min at 4 °C, and the supernatant was collected as the total protein sample. A total of 3 μL of the protein supernatant was used for BCA protein quantification. The absorbance was measured at 562 nm, and the protein concentration was calculated according to the standard curve to determine the loading amount. Subsequently, 20% volume of 5× loading buffer was added to the remaining protein sample, vortexed, and then heated in a 100 °C water bath for 5 min to denature the protein. The denatured protein sample can be used immediately for subsequent experiments or stored at −80 °C for later use.

#### 2.13.2. Western Blot Detection

Protein samples were separated using sodium dodecyl sulphate-polyacrylamide gel electrophoresis (SDS-PAGE) and transferred to a PVDF membrane for immunoassay. First, 10–15% SDS-PAGE gels were prepared. Based on the protein quantification results, 20 μg of protein was loaded onto each sample. Electrophoresis was performed at 80 V for stacking gel electrophoresis; after the protein entered the separating gel, the voltage was adjusted to 120 V and electrophoresis continued until completion. After electrophoresis, the protein was transferred from the gel to a PVDF membrane (pre-activated with methanol) at 300 mA for 100 min, all at low temperature. After transfer, the membrane was blocked with 5% skim milk blocking buffer at room temperature for 30 min. The corresponding primary antibody was then added, and the membrane was incubated overnight at 4 °C. The next day, the membrane was washed three times with TBST solution (10 min each time), followed by the addition of the corresponding HRP-labelled secondary antibody and incubation at room temperature for 2 h. After incubation, the membrane was washed three more times with TBST solution. ECL chemiluminescence reagent was then added for colour development, and protein band images were acquired using a gel imaging system. The grayscale values of the protein bands were analysed using Image Lab software (version 6.1, Bio-Rad, Hercules, CA, USA) and normalised to the internal control proteins β-actin or GAPDH.

### 2.14. Safety Evaluation

#### 2.14.1. Haemolysis Assay

To evaluate the potential damaging effect of the peptide on the erythrocyte membrane, the haemolytic activity of the peptide was detected using a horse erythrocyte haemolysis assay. Defibrinated horse blood was purchased from a commercial source. Erythrocytes were washed three times with PBS buffer (pH 7.4) to prepare a 4% (*v*/*v*) erythrocyte suspension. The peptide was first dissolved in DMSO to prepare a stock solution, and then serially diluted with PBS to working concentrations of 2–1024 μM, with the final DMSO concentration controlled below 1%. Subsequently, 100 μL of the peptide solution was mixed with 100 μL of the 4% erythrocyte suspension and incubated at 37 °C for 2 h. After incubation, the sample was centrifuged at 930× *g* for 10 min, and the supernatant was carefully transferred to a 96-well plate. The absorbance was measured at 470 nm using a Synergy HT microplate reader (BioTek, Winooski, VT, USA). Red blood cell suspension treated with 1% Triton X-100 (Sigma-Aldrich, St. Louis, MO, USA) served as a positive control, while red blood cell suspension treated with 1% DMSO (diluted with PBS) served as a negative control. Haemolysis rate was calculated using the following formula:

Haemolysis = (s − n)/(p − n)

where ‘s’ represents the optical density value of the experimental group, ‘n’ represents the optical density value of the negative control, and ‘p’ represents the optical density value of the positive control.

#### 2.14.2. MTT Cell Proliferation Inhibition Assay

Cell viability was assessed using the MTT assay. Cells in the logarithmic growth phase were seeded into 96-well plates at a density of 8 × 10^3^ cells/well and incubated for 24 h under standard culture conditions. The cells were then treated with the peptides for an additional 24 h. Subsequently, the medium was removed and MTT solution prepared in serum-free medium was added to each well, followed by incubation for 4 h at 37 °C. After incubation, the supernatant was carefully removed, and 100 μL of DMSO was added to each well to dissolve the formazan crystals. The plates were shaken for 10 min at room temperature. Absorbance was measured at 570 nm using a microplate reader (Thermo Fisher Scientific, Waltham, MA, USA). Cell viability was expressed as a percentage relative to the control group. Wells without cells but containing the same concentrations of compounds were used as blanks to eliminate background interference. All experiments were independently performed at least three times.

### 2.15. Statistical Analysis

All experiments were performed at least three independent times. Statistical analyses were performed using software Prism 9 (GraphPad Software, San Diego, CA, USA). Differences among multiple groups were analysed using one-way analysis of variance (ANOVA) followed by Fisher’s least significant difference (LSD) post hoc test. The data points are the mean of the independent experiments, and the error bar represents the standard error of the mean (SEM). Ns represents a non-significant difference; * is *p* < 0.05; ** is 0.001 < *p* < 0.01; *** is 0.0001 < *p* < 0.001; and **** is *p* < 0.0001.

## 3. Results

### 3.1. Rational Structural Modification and Physicochemical Property Analysis

**Figure 1:**
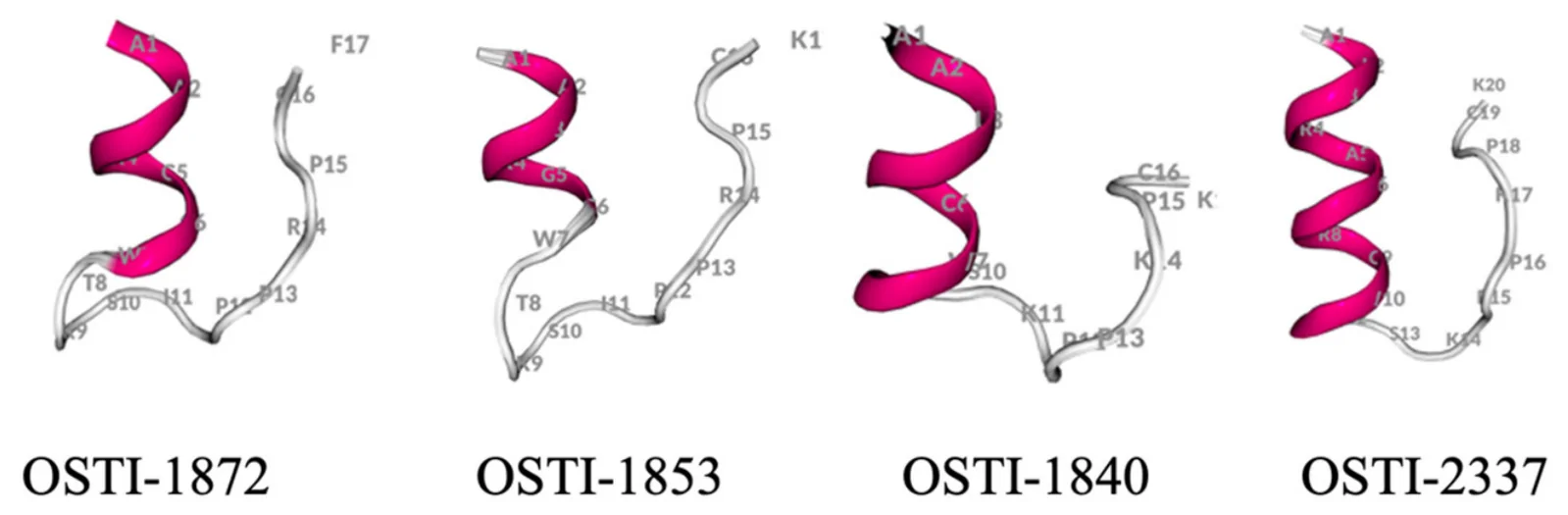
The secondary structure predicted by the online server PEP-FOLD3.5 in an aqueous environment. The α-helical structures are shown in red and random coil conformations in white.

The parent peptide, OSTI-1872, a Bowman–Birk-type trypsin inhibitor, was previously identified from the skin secretion of *Odorrana schmackeri* [18], was selected as the structural scaffold for further optimisation. Considering the conserved BBI-type inhibitory framework and the potential influence of peptide physicochemical properties on biological activity, analogue design was performed based on three rational considerations: (i) preservation of the highly conserved BBI loop responsible for maintaining the characteristic structural features of the parent peptide; (ii) modulation of peptide charge distribution to enhance peptide–cell interaction potential; and (iii) optimisation of the balance between hydrophilicity, hydrophobicity, and amphipathic characteristics rather than simply increasing the number of basic residues. Accordingly, a series of OSTI-1872 analogues were generated through targeted amino acid substitution, motif insertion, and sequence extension strategies, and their physico-chemical properties (Table 1) and secondary structures (Figure 1) were subsequently analysed.

Specifically, OSTI-1853 was obtained by replacing the C-terminal Phe (F) residue of OSTI-1872 with Lys (K). This substitution introduced an additional positive charge while reducing the hydrophobicity of the peptide terminus, resulting in an increase in net charge from 3.91 to 4.91 and enhanced hydrophilicity. Subsequently, OSTI-1840 was designed based on the OSTI-1853 sequence by replacing the IPPR motif with KPPK. This modification introduced additional positively charged residues into the central region of the peptide and altered the local charge distribution while maintaining the conserved BBI-type structural framework. As a result, OSTI-1840 exhibited further increases in net positive charge and hydrophilicity. Finally, OSTI-2337 was generated by extending the N-terminus of OSTI-1872 with an ALKR sequence. This modification further increased the overall positive charge and adjusted the physicochemical properties of the peptide, resulting in a net charge of 8.91 and a hydrophilicity value of 0.70.

### 3.2. Peptide Mass Spectrometry Identification

**Figure 2:**
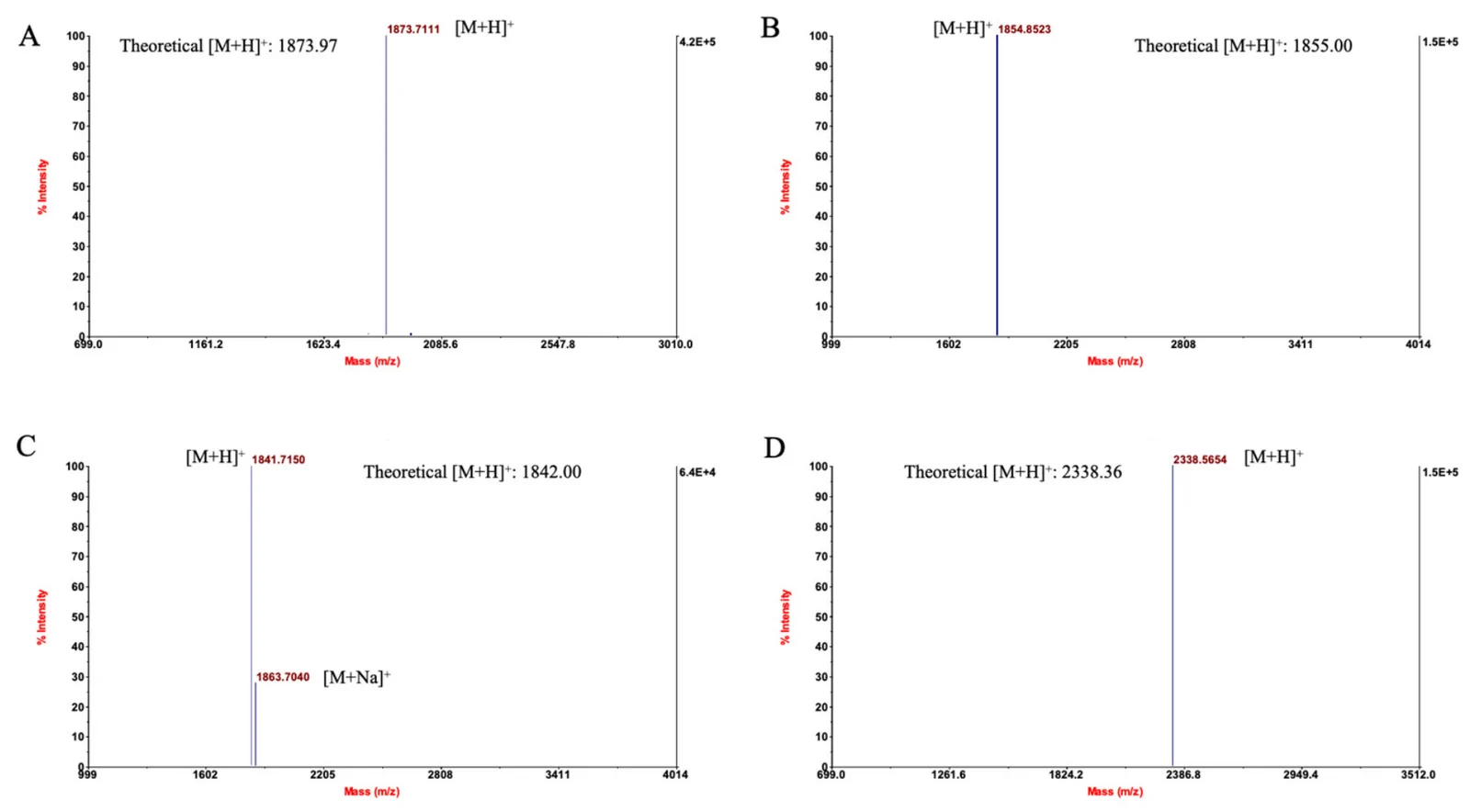
MALDI-TOF MS spectrometry of the four synthetic peptides: (**A**) OSTI-1872, (**B**) OSTI-1853, (**C**) OSTI-1840, and (**D**) OSTI-2337.

The molecular weight of the synthesised product was analysed using matrix-assisted laser desorption/ionisation time-of-flight (MALDI-TOF) mass spectrometry to verify the successful synthesis of the peptide. The measured masses (Figure 2) were in good agreement with the theoretical molecular weights calculated from the amino acid sequences, with no significant mass deviations or interference from other peaks. This result clearly indicates that the amino acid residues of the target peptide were accurately linked according to the designed sequence, and that no significant fragment deletions, abnormal modifications, or side reactions occurred during the synthesis process. The target peptide with the expected structure was successfully obtained.

### 3.3. Circular Dichroism (CD) Spectroscopy

**Figure 3:**
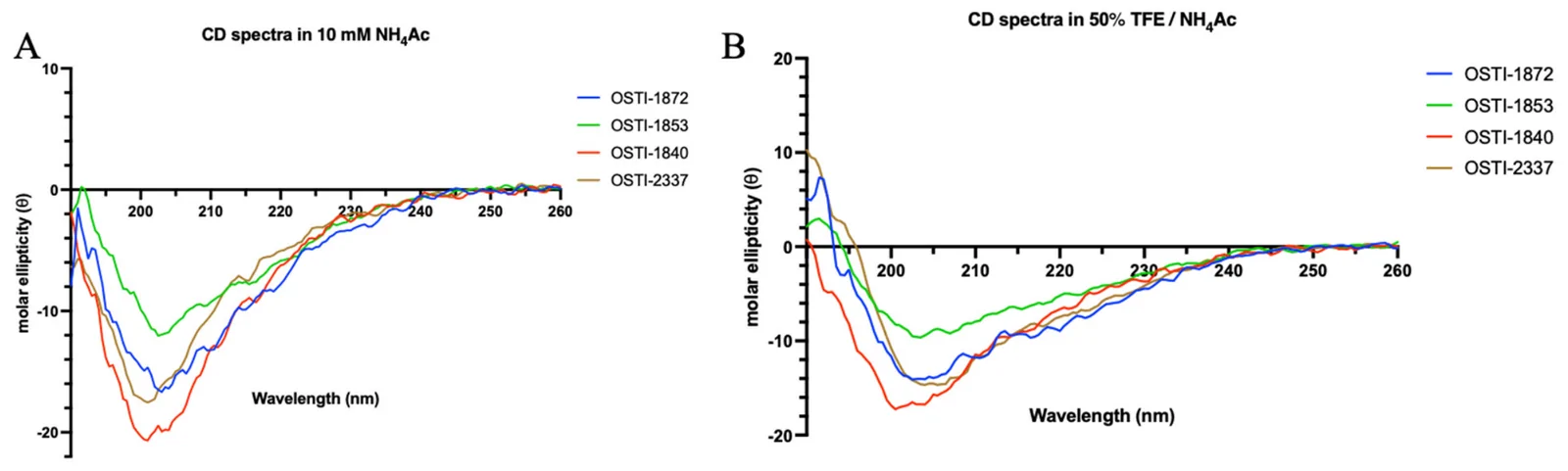
The CD spectra of OSTI−1872 and its analogues were recorded in two different environments: (**A**) a 10 mM NH_4_Ac buffer that acts as an aqueous environment and (**B**) a 50% TFE/NH_4_Ac solution which mimics a microbial membrane-mimicking hydrophobic environment.

Circular dichroism (CD) spectroscopy was used to analyse the secondary structures of OSTI-1872 and its analogues in a membrane-mimicking hydrophobic simulation solution composed of 10 mM ammonium acetate (NH_4_Ac) aqueous solution and 50% trifluoroethanol (TFE)/10 mM NH_4_Ac (Figure 3). The results showed that all peptides exhibited a mixed conformation of α-helices, β-sheets, and random coils in both solution systems. However, in the 50% TFE membrane-mimicking hydrophobic simulation environment, the peptides tended to form α-helical structures. CD spectroscopy results further indicated that in the 50% TFE/NH_4_Ac solution, each peptide showed a characteristic positive peak near 192 nm and typical negative peaks at 208 nm and 222 nm, which are characteristic spectral signals of the α-helical structure. In contrast, in a purely aqueous system, the characteristic α-helical signals of the peptides are significantly attenuated, indicating a more relaxed secondary structure predominantly adopting a random coil conformation.

### 3.4. Antimicrobial Activity Assay

The antimicrobial activities of the parent peptide OSTI-1872 and its analogues were evaluated against a panel of Gram-negative bacteria, Gram-positive bacteria, and fungi by determining their minimum inhibitory concentration (MIC) and minimum bactericidal concentration (MBC) values (Table 2). Overall, all peptides exhibited a preference for Gram-negative bacteria, while showing limited or negligible activity against Gram-positive bacteria and the tested fungal strain.

Among the tested strains, the parent peptide OSTI-1872 displayed relatively weak antimicrobial activity, with MIC values of 128 μM against *Escherichia coli* (ATCC CRM 8739) and 256 μM against *Pseudomonas aeruginosa* (ATCC CRM 9027). In addition, there were no detectable inhibitory effects against Gram-positive bacteria or *Candida albicans* (ATCC 10231) under the tested conditions. In contrast, the modified peptides exhibited improved antimicrobial activity, particularly against Gram-negative bacteria. OSTI-1840 and OSTI-2337 demonstrated the most potent activity against *E. coli* (ATCC CRM 8739), with MIC values of 16 μM, representing an approximately 8-fold enhancement compared to the parent peptide. Against Pseudomonas aeruginosa, moderate activity was observed for all analogues, with MIC values ranging from 64 to 128 μM, indicating an improvement compared to OSTI-1872. For Gram-positive bacteria, most peptides showed no significant activity (MIC > 512 μM). An exception was observed for methicillin-resistant *Staphylococcus aureus* (MRSA), where OSTI-1853 and OSTI-2337 displayed moderate activity, with MIC values of 128 μM and 64 μM, respectively. Taken together, these results indicate that structural modification of OSTI-1872 significantly enhances its antimicrobial potency against Gram-negative bacteria, while activity against Gram-positive bacteria and fungi remains limited.

### 3.5. Peptides Promote NO Production and Improve Endothelial Function

In addition to their antimicrobial activity, increasing evidence suggests that antimicrobial peptides may exhibit multiple biological functions, including anti-inflammatory and cytoprotective effects. Considering that endothelial dysfunction is closely related to oxidative stress and inflammatory damage, it was further investigated whether these peptides could exert protective effects on vascular endothelial cells. An MGO-induced HUVEC endothelial dysfunction model was established accordingly to evaluate the potential of these peptides to modulate NO production and improve endothelial function.

#### 3.5.1. Establishment of an MGO-Induced HUVEC Endothelial Dysfunction Model

NO production was used as a key indicator of endothelial function in HUVECs. To establish an in vitro endothelial dysfunction model, cells were exposed to increasing concentrations of MGO for 24 h, and NO levels in the culture supernatant were subsequently measured.

**Figure 4:**
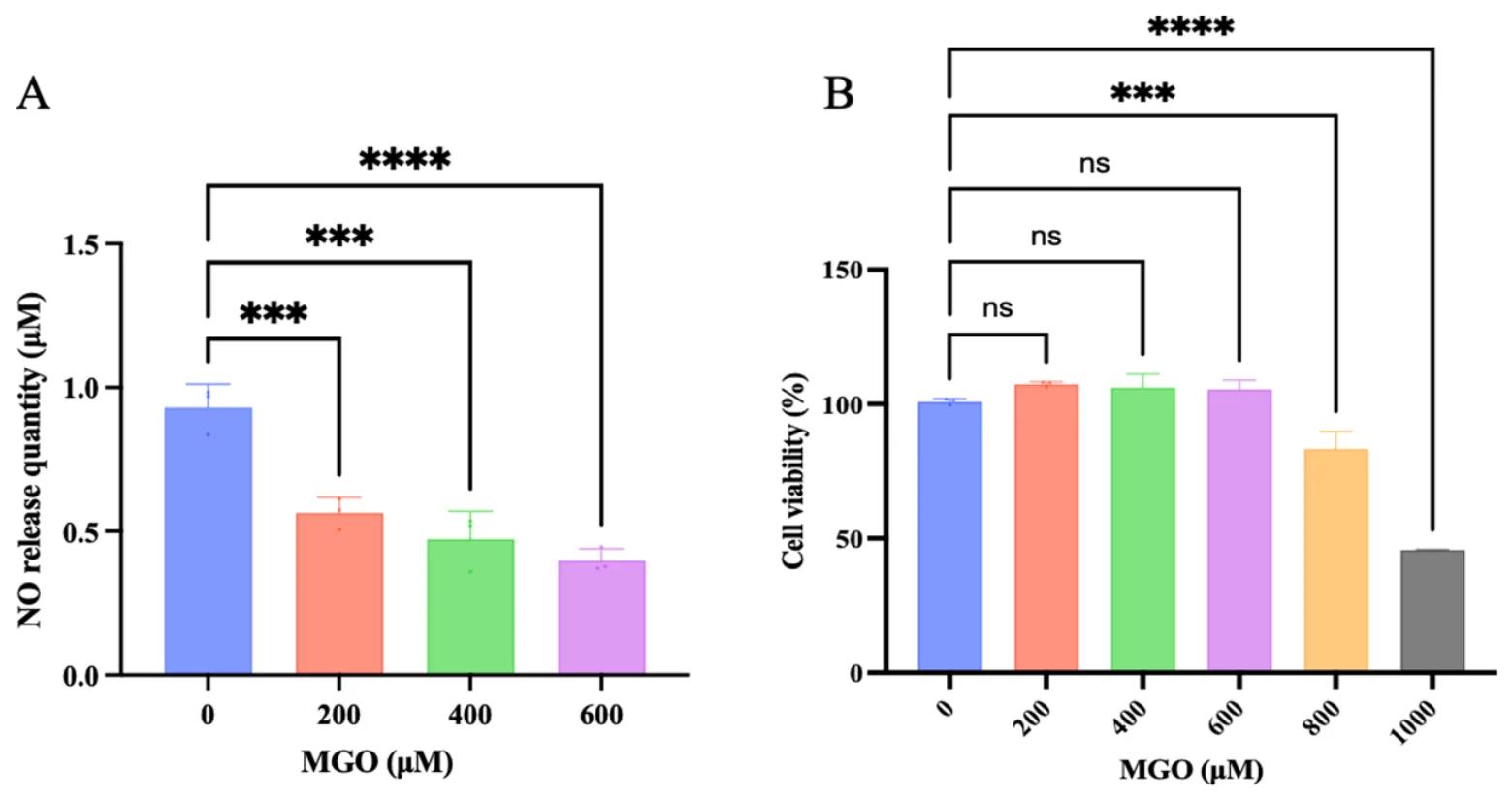
Establishment of the Vascular Dysfunction Model of HUVECs. (**A**) Effects of increasing concentrations of MGO on nitric oxide (NO) production in HUVECs. (**B**) Effect of different concentrations of MGO on HUVEC viability. Values are expressed as mean ± SEM (*n* = 3). ns represents a non-significant difference; *** *p* < 0.001, **** *p* < 0.0001.

As shown in Figure 4, NO production decreased in a concentration-dependent manner following MGO treatment. A significant reduction in NO levels was observed at 600 μM MGO, indicating marked impairment of endothelial function (Figure 4A). In parallel, cell viability was assessed to ensure that the observed reduction in NO was not solely due to excessive cytotoxicity. As presented in Figure 4B, HUVEC viability remained relatively stable at lower MGO concentrations but declined significantly when the concentration exceeded 600 μM. Based on these results, 600 μM MGO was selected as the optimal condition for inducing endothelial dysfunction, as it caused a pronounced decrease in NO production while maintaining an acceptable level of cell viability. This condition was therefore used for subsequent functional assays.

#### 3.5.2. Peptides Restoration of NO Secretion in MGO-Induced HUVECs

To evaluate the protective effects of OSTI-1872 and its analogues against MGO-induced endothelial dysfunction, NO production was assessed in HUVECs following treatment with different peptides at three concentrations (0.1, 1, and 10 μM).

**Figure 5:**
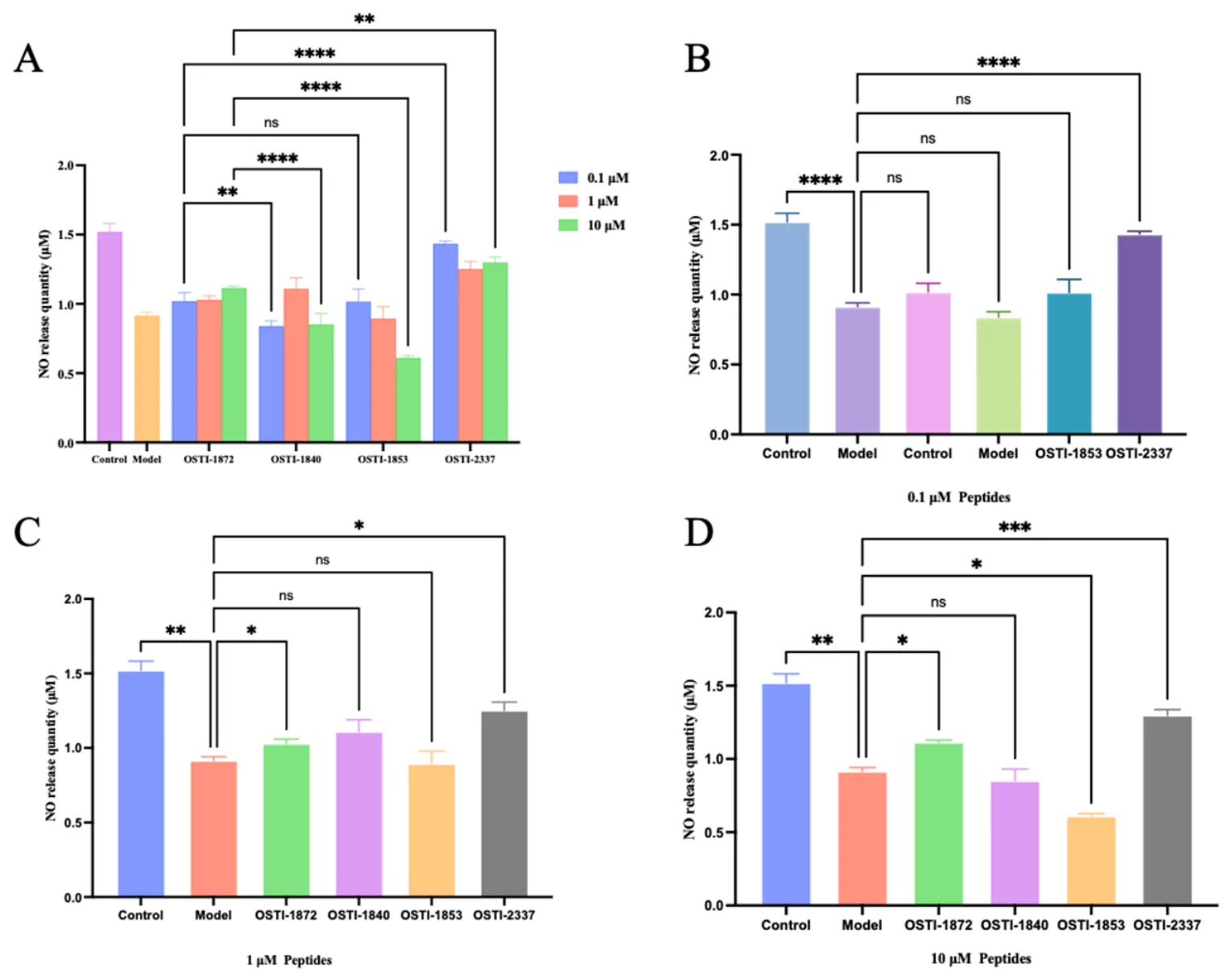
Effects of OSTI-1872 and its analogues on endothelial function in MGO-treated HUVECs. (**A**) Concentration-dependent effects of OSTI-1872 and its analogues on NO production. Comparison of NO production among different peptides at 0.1 μM (**B**), 1 μM (**C**), and 10 μM (**D**). Values are expressed as mean ± SEM (*n* = 3). ns represents a non-significant difference, * *p* < 0.05, ** *p* < 0.01, *** *p* < 0.001, **** *p* < 0.0001.

As shown in Figure 5A, MGO stimulation markedly reduced NO production compared with the control group. Treatment with OSTI-1872 and its analogues restored NO levels to varying extents, although the efficacy differed among the peptides and concentrations. To facilitate comparison, NO production was further analysed separately at each concentration (Figure 5B–D). At 0.1 μM, OSTI-2337 produced the greatest increase in NO production, showing a significantly stronger effect than both the MGO-treated model group and the parent peptide OSTI-1872. At 1 μM and 10 μM, the protective effects of the peptides remained evident, although no further enhancement over that observed at 0.1 μM was detected. Overall, these findings indicate that structural modification improved the ability of OSTI-2337 to restore NO bioavailability under MGO-induced oxidative stress, suggesting enhanced protection against endothelial dysfunction. Based on the NO production results, OSTI-1872 and OSTI-2337 were used at 0.1 μM in this and the subsequent functional and mechanistic experiments, as increasing the peptide concentration to 1 or 10 μM did not produce further improvement.

#### 3.5.3. *Peptides Restore VEGF Expression and eNOS Phosphorylation*

**Figure 6:**
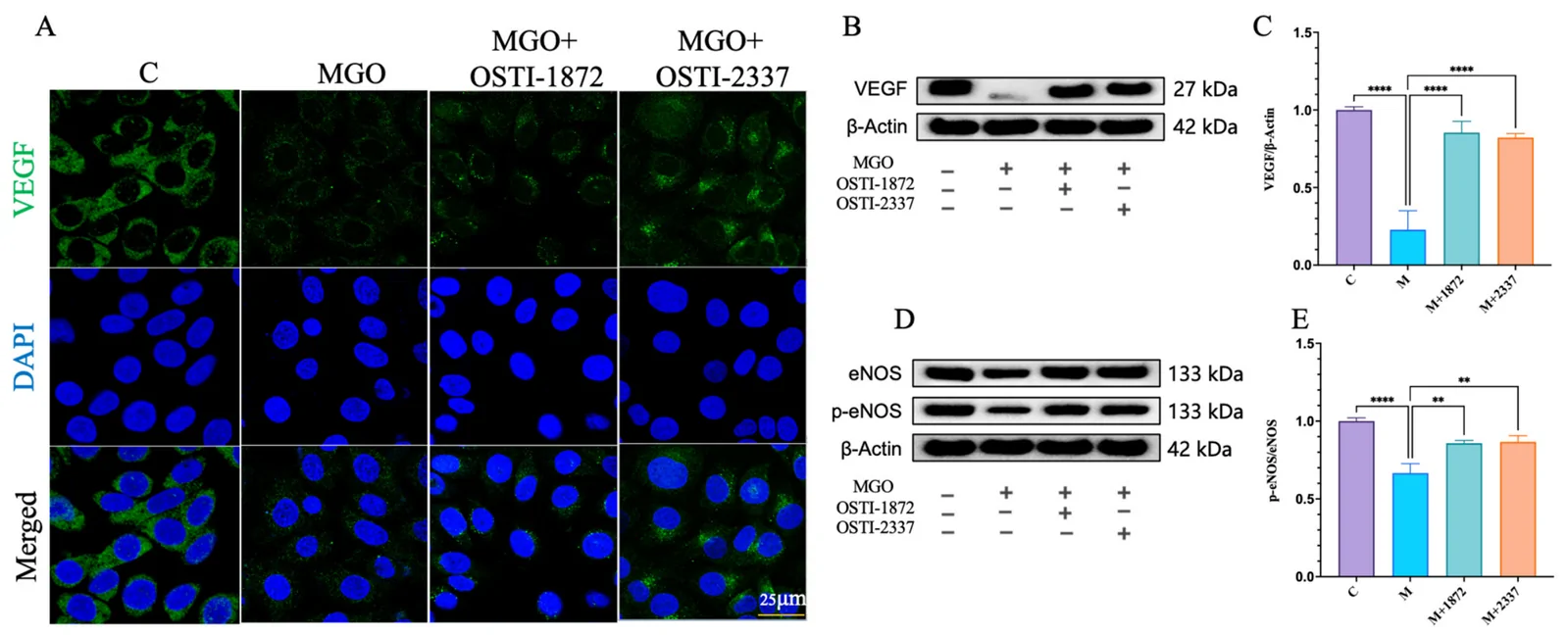
The effects of parent peptide OSTI-1872 and modified peptide OSTI-2337 on related proteins. (**A**) Immunofluorescence analysis was used to show the VEGF in HUVECs, green fluorescence represents VEGF immunostaining and blue fluorescence indicates DAPI-stained nuclei. (**B**–**E**) Western blotting analysis for VEGF, p-eNOS, and eNOS. The band density of proteins was analysed with ImageJ software (Fiji/ImageJ, version 1.54). Values are expressed as mean ± SEM (*n* = 3). ** *p* < 0.01, **** *p* < 0.0001. Original Western blot images can be found in Supplementary Materials.

Immunofluorescence staining and Western blot analysis were used to evaluate VEGF protein levels and the phosphorylation status of endothelial nitric oxide synthase (eNOS). As shown in Figure 6A, compared with the control group, the VEGF immunofluorescence signal (green) in HUVECs was markedly reduced following MGO treatment, indicating suppression of angiogenesis-related protein levels. Treatment with the parent peptide OSTI-1872 and the modified peptide OSTI-2337 significantly increased VEGF fluorescence intensity, with a more pronounced recovery observed in the OSTI-2337 group. Western blot analysis further confirmed these findings (Figure 6B). Compared with the control group, MGO treatment significantly decreased VEGF protein levels and the p-eNOS/eNOS ratio. In contrast, both OSTI-1872 and OSTI-2337 treatments significantly restored VEGF protein levels and increased the p-eNOS/eNOS ratio. Quantitative analysis showed that OSTI-2337 exerted a slightly stronger effect than the parent peptide at the same concentration. These results indicate that both peptides can attenuate MGO-induced endothelial dysfunction, as reflected by the restoration of VEGF protein levels and eNOS phosphorylation.

### 3.6. The Restoration of Angiogenic Capacity in MGO-Induced HUVEC Injury Model

The angiogenic capacity of HUVECs was evaluated using a tube formation assay on Matrigel. Based on its superior performance in restoring NO production compared with the parent peptide, OSTI-2337 was selected for further functional and mechanistic studies alongside OSTI-1872.

**Figure 7:**
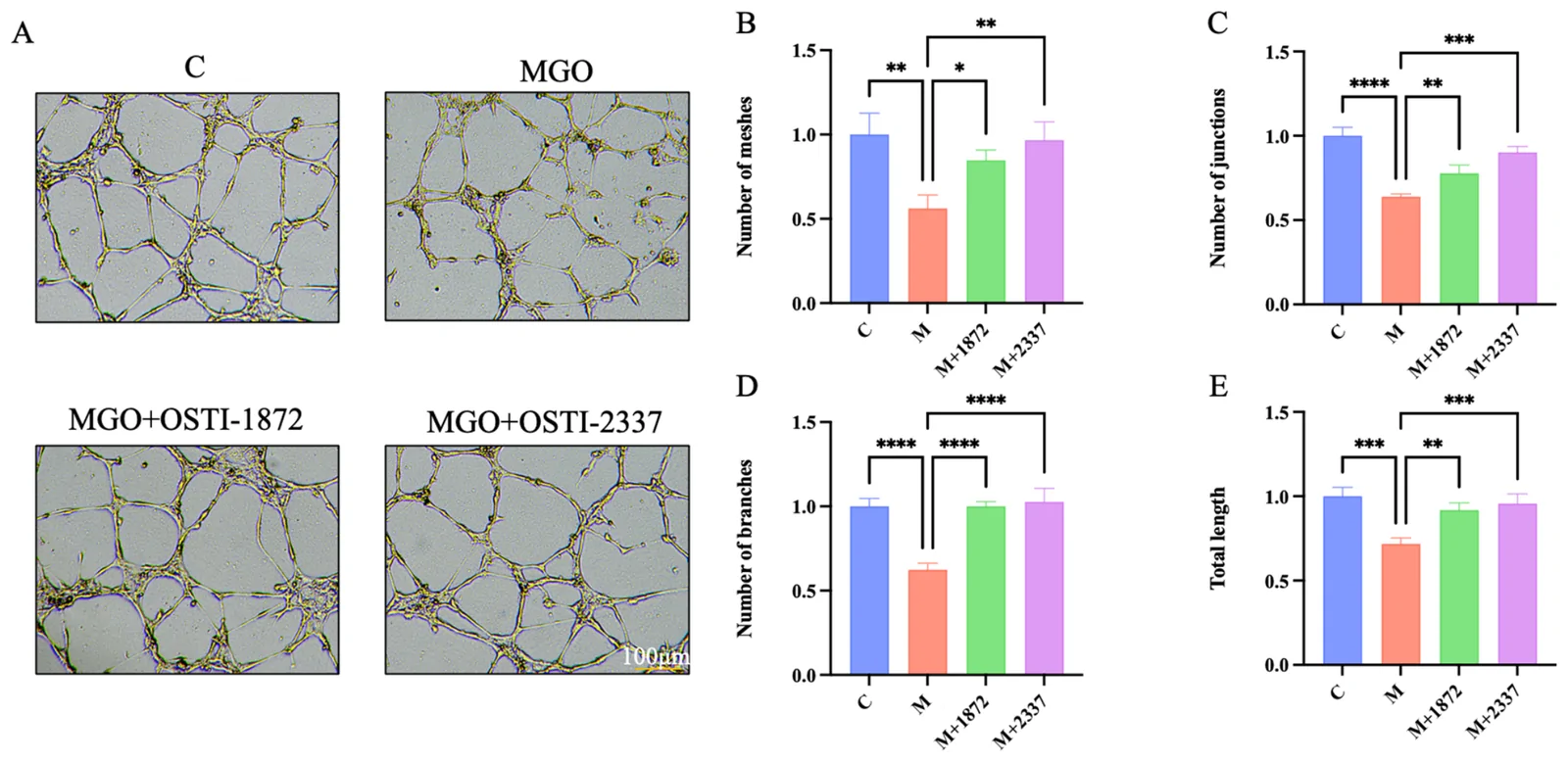
Effects of parent peptide OSTI-1872 and modified Peptide OSTI-2337 on tube formation ability of HUVECs. (**A**) Observation results of angiogenesis experiment. Peptides alleviate the reduction in meshes (**B**), junctions (**C**), branches (**D**), and total length (**E**) caused by MGO. Values are expressed as mean ± SEM (*n* = 3). * *p* < 0.05, ** *p* < 0.01, *** *p* < 0.001, **** *p* < 0.0001.

Under normal conditions, endothelial cells form a complete and tightly connected tubular network structure. However, when angiogenic capacity is impaired, the luminal structure appears sparse, broken, or incomplete. As shown in Figure 7A, compared with the control group, the luminal network formed by HUVECs after MGO treatment was significantly reduced, and the structure was sparse and fragmented, indicating that MGO successfully induced impairment of angiogenic capacity. After treatment with the parent peptide OSTI-1872 and the modified peptide OSTI-2337, the tubular network structure was significantly restored, with increased numbers of tubular structures and improved network integrity. Notably, the OSTI-2337-treated group exhibited a denser network, with morphology closer to that of the control group. Further quantitative analysis of angiogenesis-related parameters (Figure 7B–E) showed that, compared with the MGO-treated group, both peptides significantly increased the number of meshes, junctions, branches, and total tube length. Moreover, OSTI-2337 demonstrated a consistently stronger restorative effect than OSTI-1872 at the same concentration. These results indicate that OSTI-2337 more effectively attenuates MGO-induced impairment of angiogenic capacity in HUVECs.

### 3.7. Peptides Improve MGO-Induced HUVEC Migration Impairment

**Figure 8:**
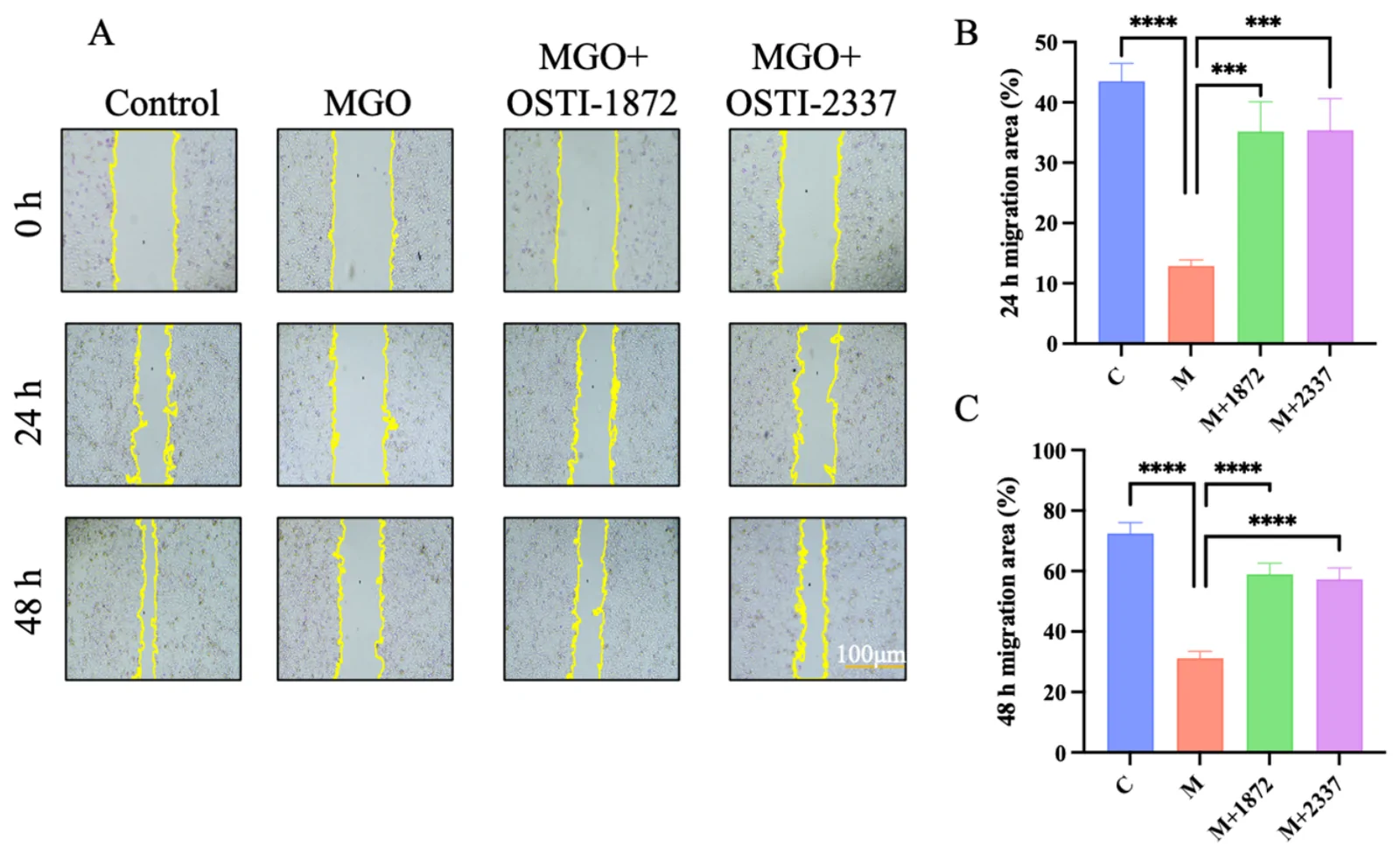
Cell scratch assay reveals parent peptide OSTI-1872 and modified peptide OSTI-2337 alleviates migration barrier caused by MGO. (**A**) Observation results of scratch experiment, yellow lines indicate the wound edges used for quantitative analysis of scratch closure and cell migration. Migration area of cells at 24 h (**B**) and 48 h (**C**). Values are expressed as mean ± SEM (*n* = 3). *** *p* < 0.001, **** *p* < 0.0001.

The effects of the metabolic peptide OSTI-1872 and the modified peptide OSTI-2337 on MGO-induced impaired HUVEC migration were evaluated using a scratch wound healing assay. Serum-free medium was used to eliminate interference from cell proliferation during the experiment. As shown in Figure 8A, compared with the control group, the degree of scratch wound healing in the MGO-treated group was significantly reduced at 24 h and 48 h, indicating that MGO treatment significantly inhibited the migration ability of HUVECs. After intervention with the metabolic peptide OSTI-1872 and the modified peptide OSTI-2337, the degree of scratch wound healing was significantly increased, indicating that both peptides can promote the recovery of the migration ability of damaged endothelial cells. Further quantitative analysis of the migration area (Figure 8B,C) showed that, compared with the MGO model group, both the OSTI-1872 and OSTI-2337 treatment groups significantly increased the cell migration area, indicating an attenuation of the MGO-induced impairment in migration ability. These results suggest that both the parent peptide OSTI-1872 and the modified peptide OSTI-2337 can alleviate MGO-induced impairment of migration in HUVECs.

### 3.8. Peptides Alleviate MGO-Induced Oxidative Stress

#### 3.8.1. Peptides Reduce Intracellular ROS Levels

**Figure 9:**
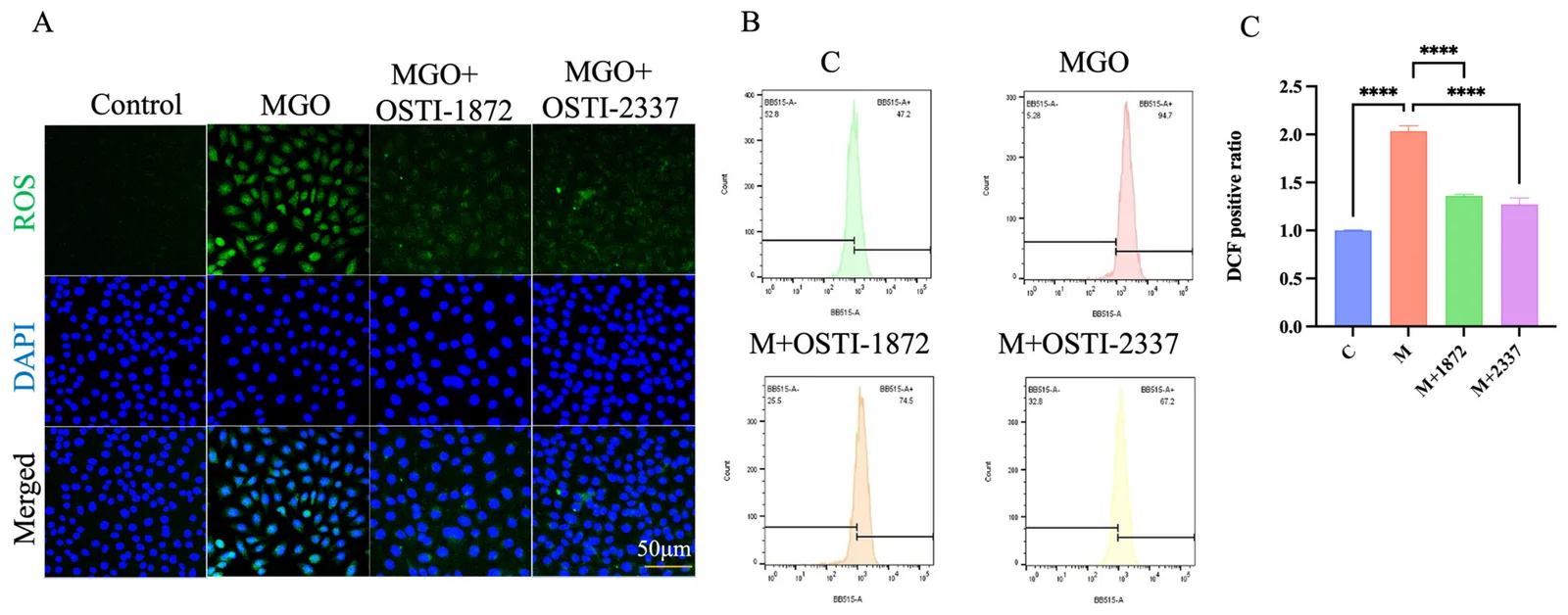
Parent peptide OSTI-1872 and modified peptide OSTI-2337 reduces the release of reactive oxygen species (ROS). (**A**) Fluorescence changes in reactive oxygen species detected by DCFH-DA, green fluorescence represents ROS signals and blue fluorescence indicates DAPI-stained nuclei. (**B**) ROS level was measured by DCF fluorescence by flow cytometry and data were analysed by Flow jo (**C**). Values are expressed as mean ± SEM (*n* = 3). **** *p* < 0.0001.

To evaluate the effects of the parent peptide OSTI-1872 and the modified peptide OSTI-2337 on MGO-induced oxidative stress, intracellular ROS levels in HUVECs were measured using the DCFH-DA fluorescent probe. As shown in Figure 9, compared with the control group, the green fluorescence signal in the MGO-treated group was significantly enhanced, indicating increased intracellular ROS levels. The green fluorescence represents the oxidised product (DCF) generated from DCFH in the presence of ROS. After treatment with OSTI-1872 and OSTI-2337, the fluorescence intensity was significantly reduced, suggesting decreased ROS levels in HUVECs. Further quantitative analysis of intracellular ROS levels was performed using flow cytometry. The results showed that, compared with the MGO-treated group, both OSTI-1872 and OSTI-2337 significantly decreased ROS levels, indicating attenuation of MGO-induced oxidative stress. These results suggest that both peptides can alleviate oxidative stress in MGO-treated HUVECs.

#### 3.8.2. Peptides Improvement of Oxidative Stress-Related Indicators

**Figure 10:**
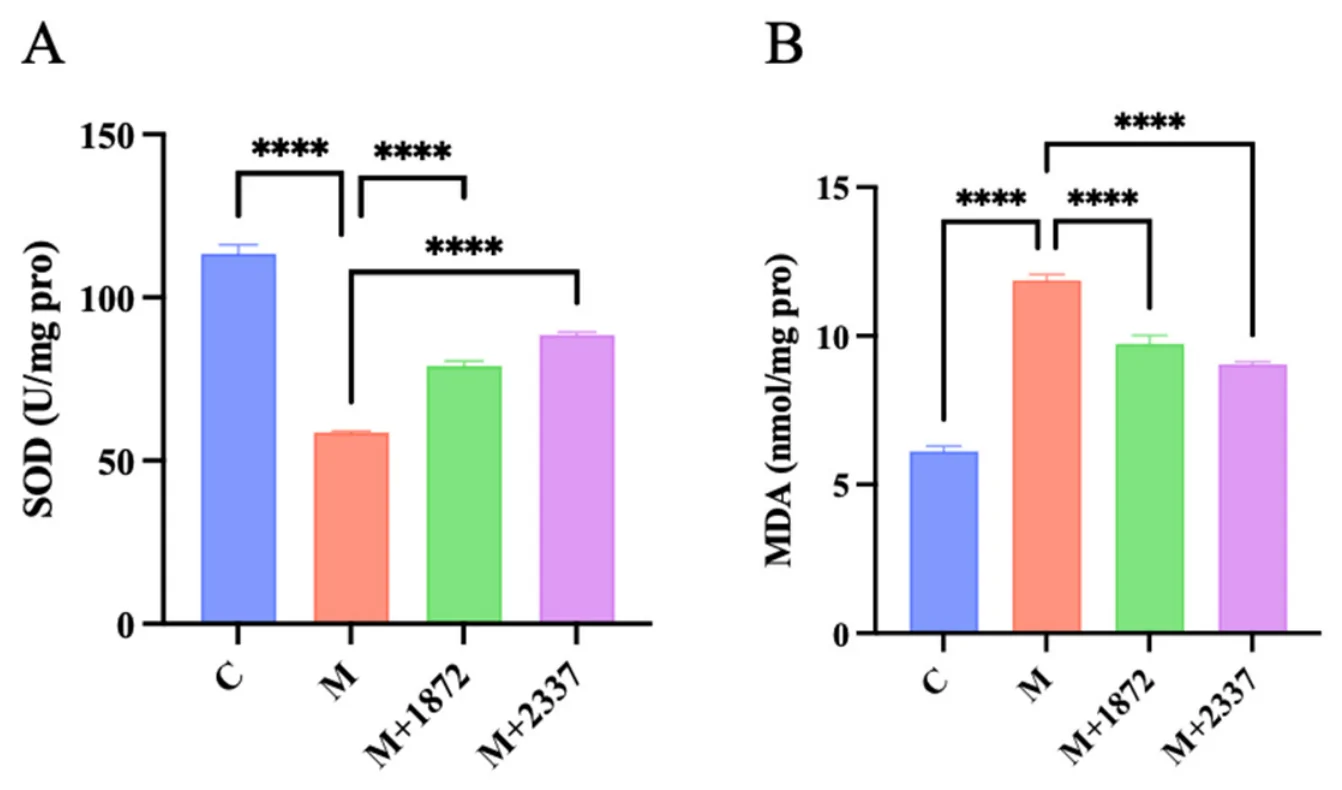
Parent peptide OSTI-1872 and modified peptide OSTI-2337 increases (**A**) the activity of superoxide dismutase (SOD) and reduces (**B**) the mass of malondialdehyde (MDA). Values are expressed as mean ± SEM (*n* = 3). **** *p* < 0.0001.

To further evaluate the regulatory effects of the parent peptide OSTI-1872 and the modified peptide OSTI-2337 on MGO-induced oxidative stress, intracellular superoxide dismutase (SOD) activity and malondialdehyde (MDA) content were measured. As shown in Figure 10A, compared with the MGO-treated group, SOD activity was significantly increased in both peptide-treated groups. Meanwhile, MDA levels (Figure 10B), an indicator of lipid peroxidation, were significantly reduced following peptide treatment. These results suggest that both OSTI-1872 and OSTI-2337 can improve cellular redox status and attenuate MGO-induced oxidative stress in HUVECs.

#### 3.8.3. Peptides Alleviate DNA Oxidative Damage

**Figure 11:**
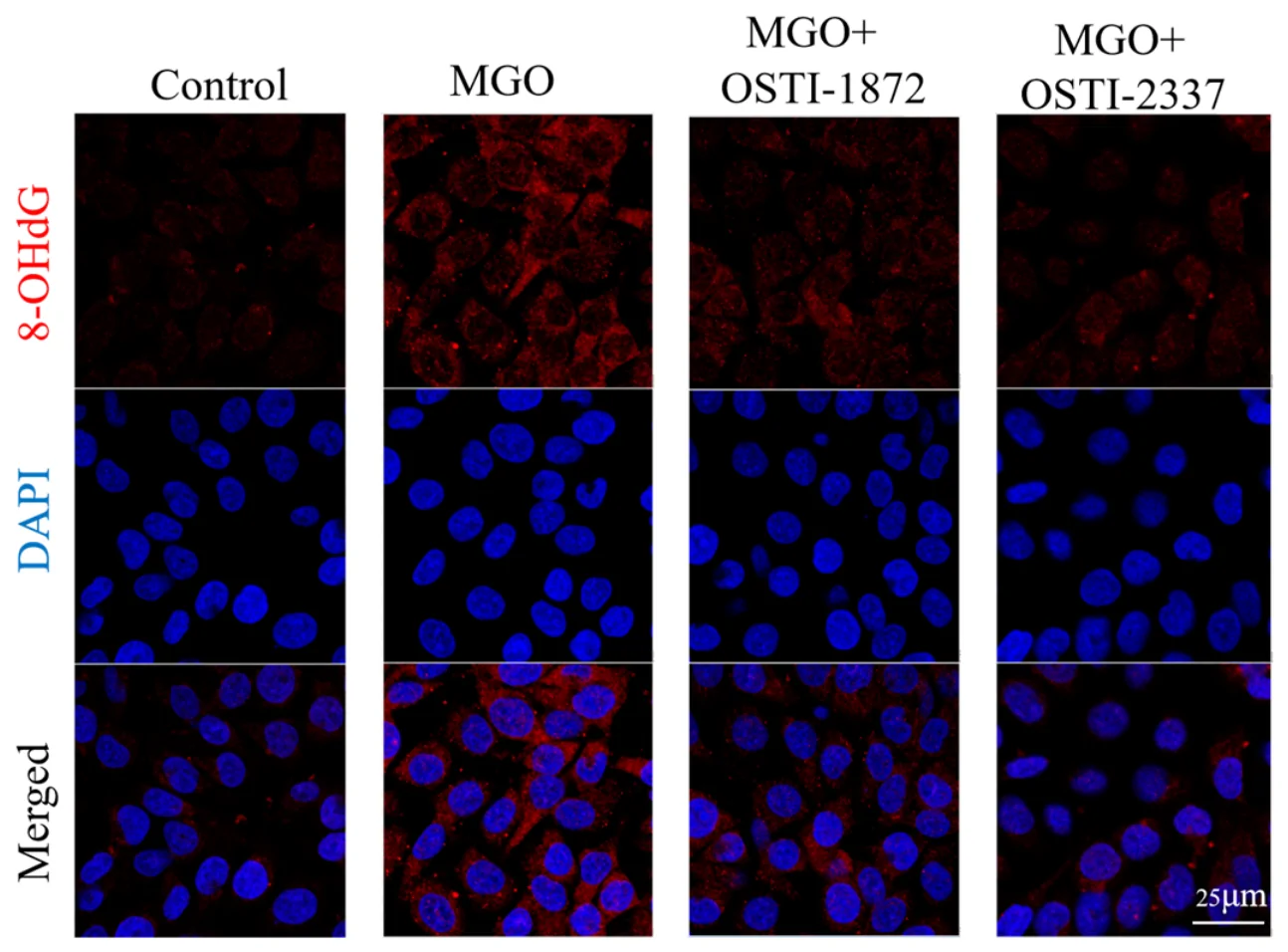
Representative immunofluorescence images showing the effect of the parent peptide OSTI-1872 and the modified peptide OSTI-2337 on MGO-induced oxidative DNA damage in HUVECs. 8-OHdG was immunostained with specific antibodies (red), and nuclei were counterstained with DAPI (blue).

To further evaluate the DNA damage caused by MGO-induced oxidative stress, immunofluorescence staining was used to detect the expression level of intracellular 8-hydroxydeoxyguanosine (8-OHdG), a commonly used biomarker for DNA oxidative damage. As shown in Figure 11, compared with the blank control group, the fluorescence signal of 8-OHdG in HUVECs treated with MGO was significantly enhanced, indicating that MGO treatment induced significant DNA oxidative damage. After intervention with the parent peptide OSTI-1872 and the modified peptide OSTI-2337, the intracellular 8-OHdG fluorescence signal was significantly weakened, suggesting that both peptides could reduce the level of MGO-induced DNA oxidative damage. These results indicate that the parent peptide OSTI-1872 and the modified peptide OSTI-2337 can alleviate MGO-induced DNA oxidative damage in HUVEC.

### 3.9. Activation of the Nrf2 Antioxidant Signalling Pathway by Peptides

#### 3.9.1. Promotion of Nrf2 Protein Expression and Nuclear Translocation

**Figure 12:**
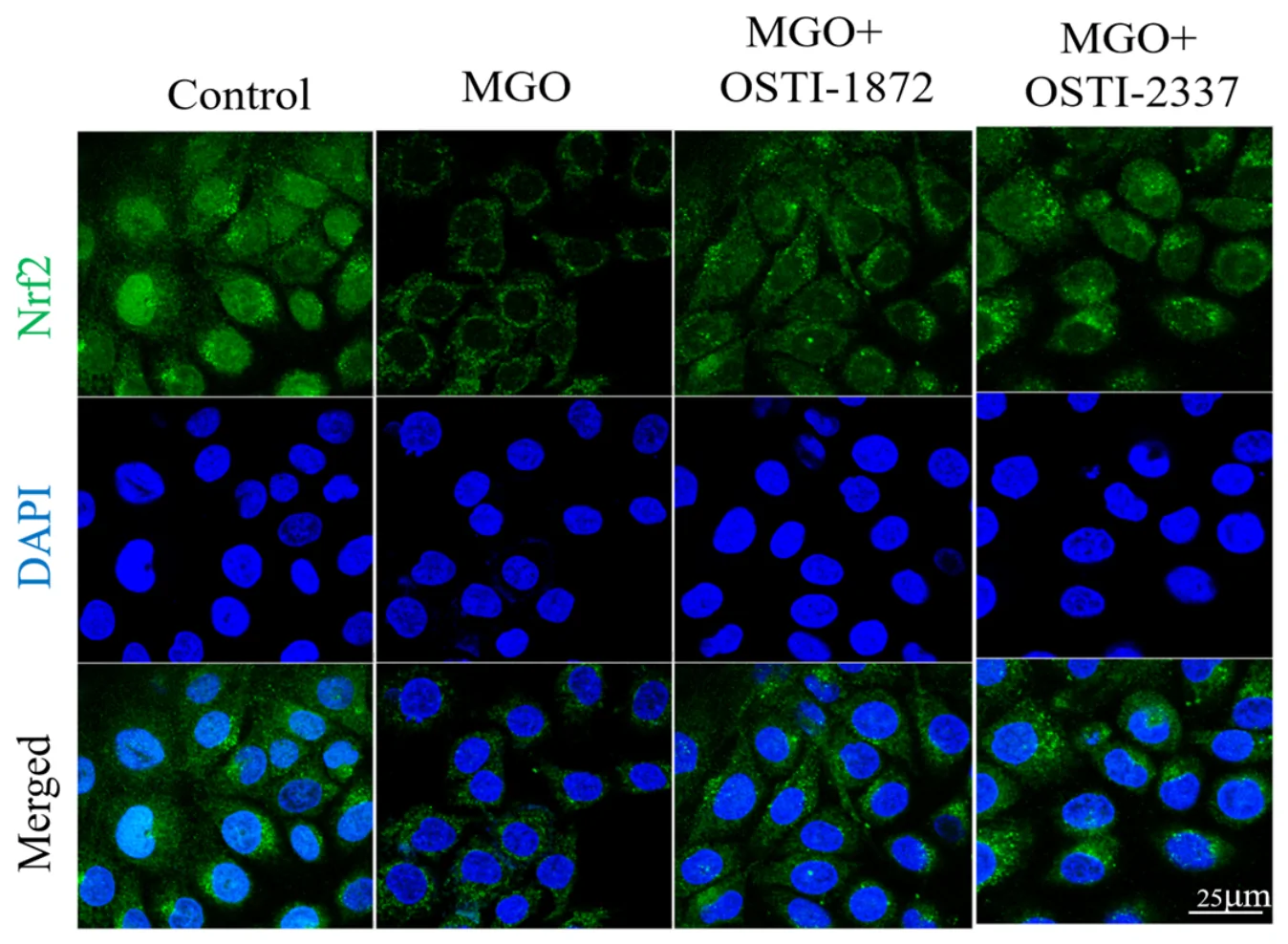
Representative immunofluorescence images showing the effect of the parent peptide OSTI-1872 and the modified peptide OSTI-2337 on Nrf2 expression and nuclear localisation in MGO-treated HUVECs. Blue: nucleus (DAPI); Green: Nrf2-staining.

To further investigate the regulatory effects of the parent peptide OSTI-1872 and the modified peptide OSTI-2337 on antioxidant signalling pathways, Nrf2 protein levels and intracellular localisation were evaluated by immunofluorescence staining. As shown in Figure 12, compared with the control group, MGO treatment markedly reduced the Nrf2 fluorescence signal in HUVECs and decreased its nuclear localisation. Following treatment with OSTI-1872 and OSTI-2337, the intracellular Nrf2 fluorescence intensity was increased, and nuclear localisation of Nrf2 was enhanced. This redistribution of Nrf2 from the cytoplasm to the nucleus is generally associated with activation of antioxidant defence pathways. These results suggest that both peptides may alleviate MGO-induced oxidative stress by modulating Nrf2-related antioxidant responses. This observation is consistent with the reduced ROS levels and improved antioxidant indicators observed in peptide-treated groups.

#### 3.9.2. Regulation of the Keap1/Nrf2 Signalling Pathway

**Figure 13:**
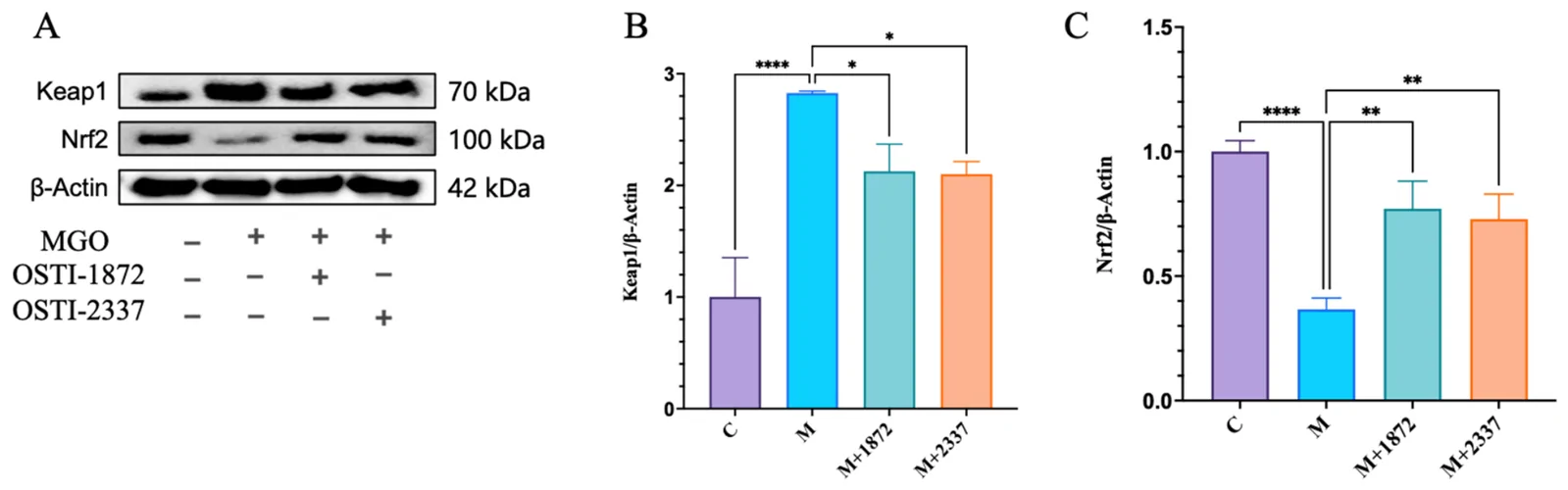
Parent peptide OSTI-1872 and modified peptide OSTI-2337 elevate the level of Nrf2 by binding to Keap1, the upstream negative regulatory protein of Nrf2. (**A**–**C**) Western blotting analysis for Keap1 and Nrf2. Values are expressed as mean ± SEM (*n* = 3). * *p* < 0.05, ** *p* < 0.01, **** *p* < 0.0001. Original Western blot images can be found in Supplementary Materials.

To further investigate the regulatory effects of the parent peptide OSTI-1872 and the modified peptide OSTI-2337 on the Nrf2 antioxidant signalling pathway, the expression levels of Keap1 and Nrf2 proteins were detected by Western blot. As shown in Figure 13A, compared with the control group, the expression of Keap1 protein was significantly increased after MGO treatment, while the level of Nrf2 protein was decreased. After intervention with the parent peptide OSTI-1872 and the modified peptide OSTI-2337, the expression of Keap1 protein was decreased, while the level of Nrf2 protein was increased significantly. Further quantitative analysis results (Figure 13B,C) showed that compared with the MGO model group, both the parent peptide OSTI-1872 and the modified peptide OSTI-2337 could significantly reduce the expression of Keap1 protein and increase the level of Nrf2 protein. The above results indicate that the parent peptide OSTI-1872 and the modified peptide OSTI-2337 can regulate the Keap1/Nrf2 signalling pathway, thereby participating in the alleviation of MGO-induced oxidative stress.

#### 3.9.3. Promotion the Expression of Downstream Antioxidant Proteins of Nrf2

**Figure 14:**
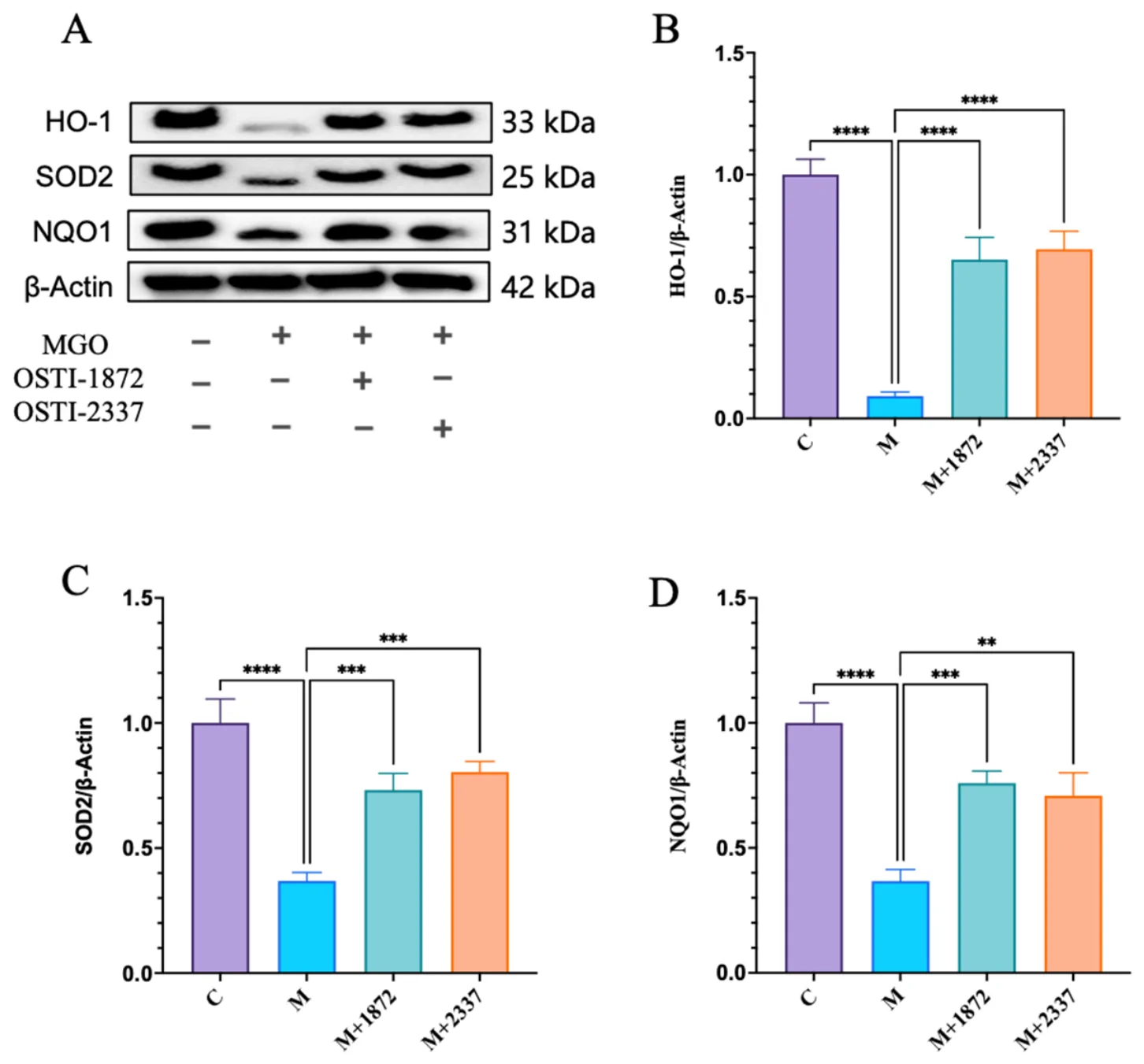
Effects of OSTI-1872 and OSTI-2337 on Nrf2-related antioxidant protein expression. (**A**–**D**) Western blot analysis of HO-1, SOD2, and NQO1. Protein band densities were quantified using ImageJ software (Fiji/ImageJ, version 1.54). Data are presented as mean ± SEM (*n* = 3). ** *p* < 0.01, *** *p* < 0.001, **** *p* < 0.0001. Original Western blot images can be found in Supplementary Materials.

To further evaluate changes in downstream antioxidant proteins associated with Nrf2 signalling, the expression levels of HO-1, SOD2, and NQO1 were analysed by Western blot. As shown in Figure 14, compared with the control group, MGO treatment significantly decreased the expression levels of HO-1, SOD2, and NQO1. Treatment with OSTI-1872 and OSTI-2337 significantly increased the protein levels of these antioxidant markers. Notably, OSTI-2337 exhibited a stronger effect than the parent peptide in restoring the expression of HO-1, SOD2, and NQO1at the same concentration. These proteins are commonly recognised as downstream targets of Nrf2 signalling. Therefore, the observed changes may be associated with modulation of Nrf2-related antioxidant responses, rather than direct regulation of individual proteins by the peptides.

### 3.10. Regulation of the PI3K/AKT/GSK3β Signalling Pathway

PI3K is a key intracellular signalling molecule, and its downstream effector AKT plays a central role in regulating endothelial function and vascular homeostasis. The PI3K/AKT pathway is known to modulate eNOS phosphorylation and VEGF expression, and AKT can negatively regulate GSK3β activity through phosphorylation. To further investigate the effects of OSTI-1872 and OSTI-2337 on MGO-induced alterations in signalling pathways, the phosphorylation levels of PI3K and AKT, as well as GSK3β expression, were analysed by Western blot.

**Figure 15:**
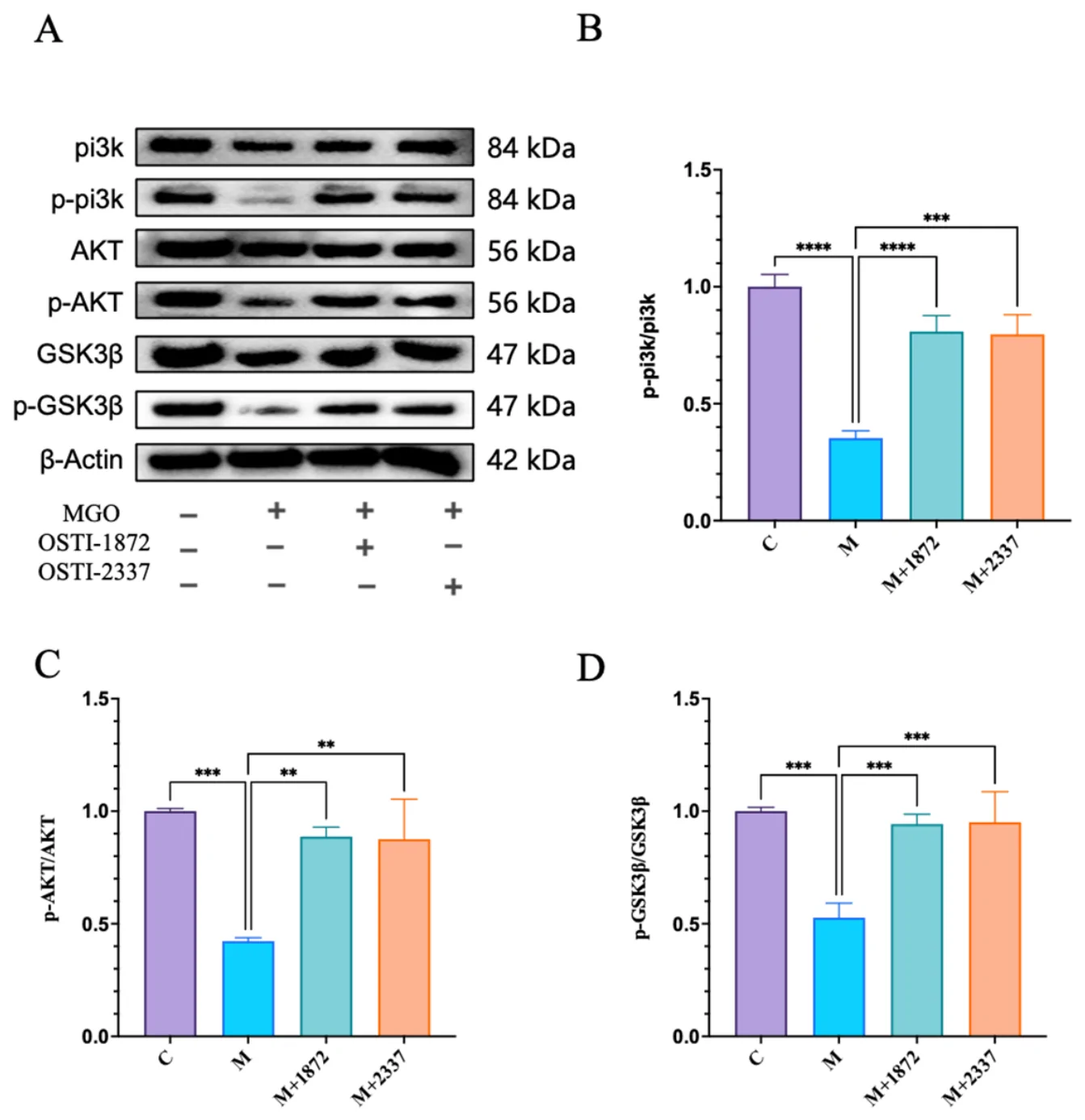
Effects of OSTI-1872 and OSTI-2337 on PI3K/AKT/GSK3β signalling in MGO-treated HUVECs. (**A**–**D**) Western blot analysis of p-PI3K, PI3K, p-AKT, AKT, p-GSK-3β, and GSK-3β. Protein band densities were quantified using ImageJ software (Fiji/ImageJ, version 1.54). Data are presented as mean ± SEM (*n* = 3). ** *p* < 0.01, *** *p* < 0.001, **** *p* < 0.0001. Original Western blot images can be found in Supplementary Materials.

As shown in Figure 15, MGO treatment markedly decreased the p-PI3K/PI3K, p-AKT/AKT, and p-GSK3β/GSK3β ratios compared with the control group. Treatment with either OSTI-1872 or OSTI-2337 significantly restored these phosphorylation ratios compared with the MGO-treated group. These results indicate that OSTI-1872 and OSTI-2337 attenuated MGO-induced suppression of the PI3K/AKT/GSK3β signalling pathway.

### 3.11. Enhancement of GLO1 Protein Expression

**Figure 16:**
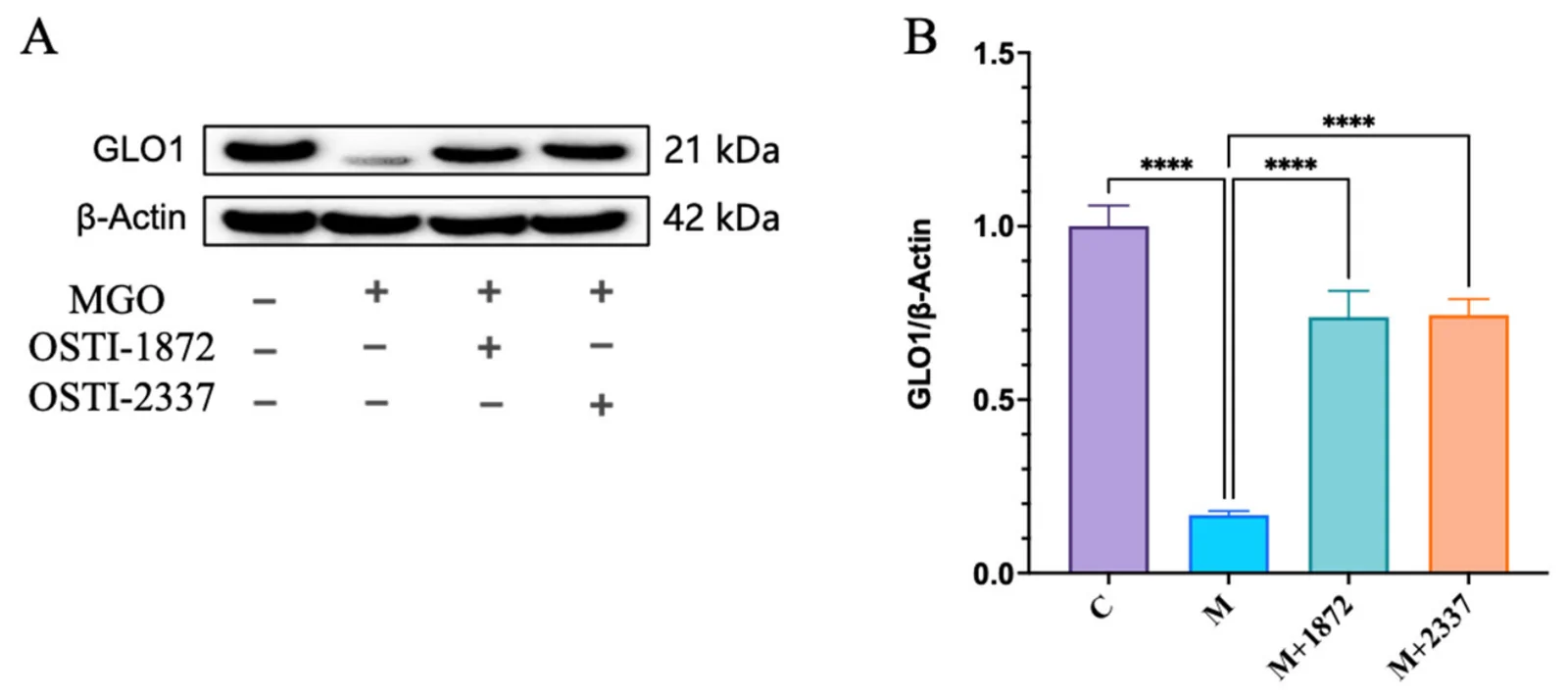
Parent peptide OSTI-1872 and modified peptide OSTI-2337 promote GLO1 expression. (**A**,**B**) Western blotting analysis for GLO1. The band density of proteins was analysed with ImageJ software (Fiji/ImageJ, version 1.54). Values are expressed as mean ± SEM (*n* = 3). **** *p* < 0.0001. Original Western blot images can be found in Supplementary Materials.

To further assess the potential involvement of peptides in MGO detoxification, the expression of glyoxalase 1 (GLO1), a key enzyme involved in MGO detoxification, was analysed by Western blot. As shown in Figure 16, MGO treatment markedly decreased GLO1 protein expression compared with the control group. Treatment with either OSTI-1872 or OSTI-2337 significantly restored GLO1 protein expression compared with the MGO-treated group. These findings suggest that both peptides may modulate the glyoxalase system in response to MGO-induced stress.

### 3.12. Biocompatibility Evaluation

#### 3.12.1. Effects of Peptides on HUVEC Viability

**Figure 17:**
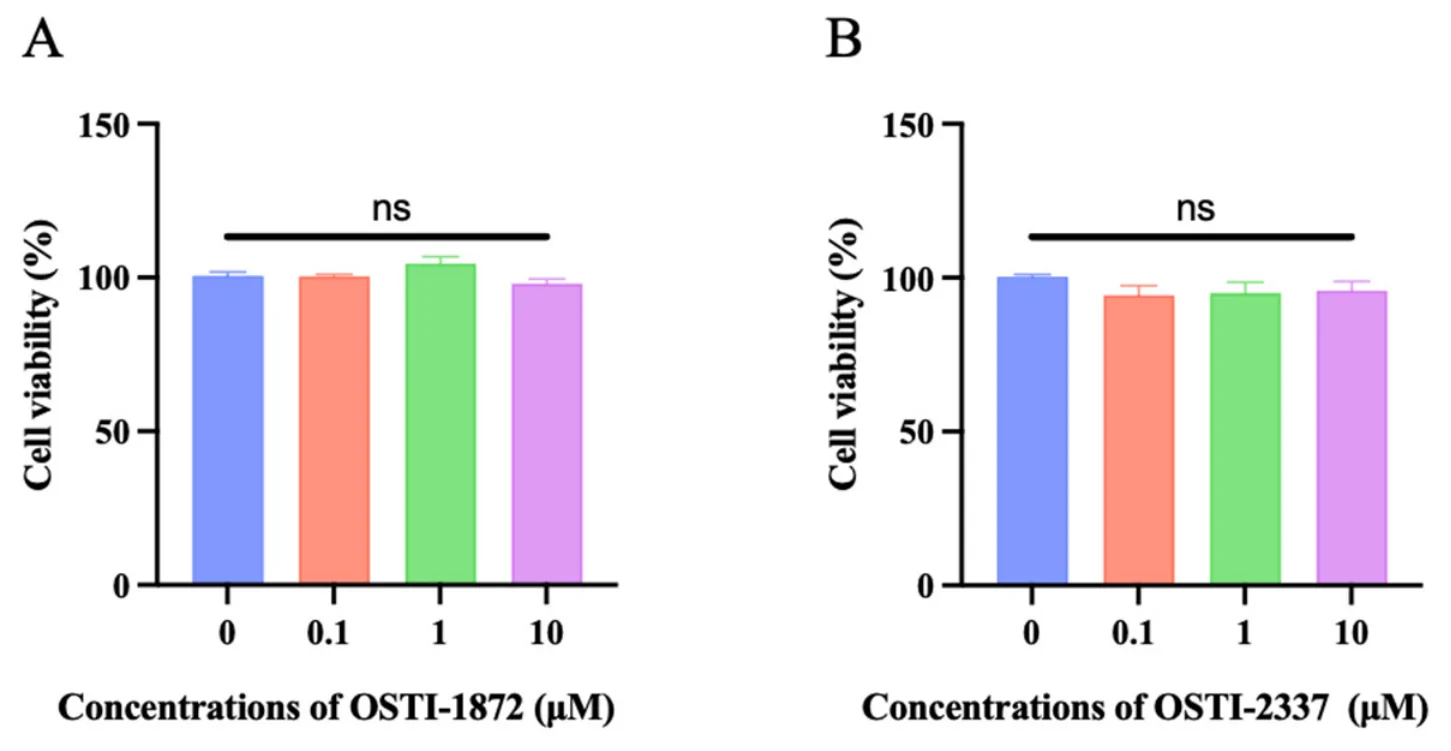
Effects of parent peptide OSTI-1872 (**A**) and modified peptide OSTI-2337 (**B**) on HUVEC viability. Values are expressed as mean ± SEM (*n* = 3). ns represents a non-significant difference.

To assess the potential cytotoxicity of the parent peptide OSTI-1872 and the modified peptide OSTI-2337 on endothelial cells, the MTT assay was used to detect the effects of different concentrations of the peptides on HUVEC viability. As shown in Figure 17, within the concentration range of 0.1–10 μM, neither the parent peptide OSTI-1872 nor did the modified peptide OSTI-2337 have a significant effect on HUVEC viability; cell viability remained at a high level, indicating that neither peptide exhibited significant cytotoxicity within this concentration range.

#### 3.12.2. Evaluation of In Vitro Haemolytic Activity

**Figure 18:**
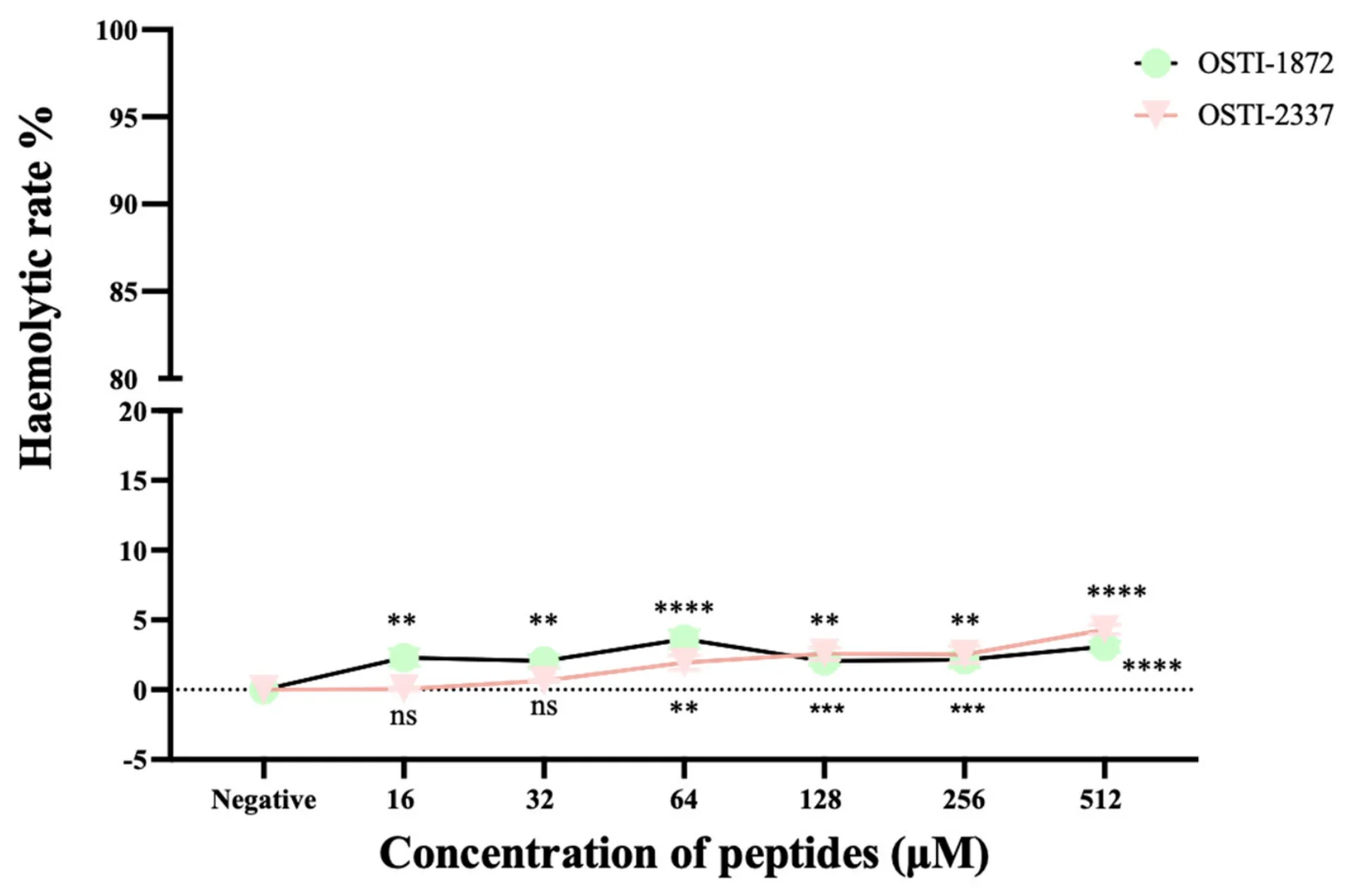
Haemolytic activity of parent peptide OSTI-1872 and modified peptide OSTI-2337 against equine erythrocytes at concentrations ranging from 1 to 512 μM. The percentage of haemolysis was calculated by comparing the effects with the positive control (1% Triton X-100) and the negative control (PBS). Values are expressed as mean ± SEM (*n* = 3). ns represents a non-significant difference; ** *p* < 0.01, *** *p* < 0.001, **** *p* < 0.0001.

To further evaluate the blood compatibility of the peptides, the haemolytic activity of the parent peptide OSTI-1872 and the modified peptide OSTI-2337 was detected using an in vitro erythrocyte haemolysis assay. As shown in Figure 18, within the tested concentration range, neither peptide induced a significant erythrocyte haemolytic reaction, with haemolysis rates below 10%. Even at the highest tested concentration, both the parent peptide and the modified peptide maintained low haemolytic levels, indicating good blood compatibility.

## 4. Discussion

Amphibian-derived peptides have attracted increasing attention because of their diverse biological activities, particularly in anti-infective and anticancer research [19,20]. However, their potential relevance to vascular injury, especially diabetes-associated endothelial dysfunction, remains insufficiently explored. Endothelial dysfunction is a central pathological event in diabetic vascular complications and chronic wound repair, where impaired nitric oxide bioavailability, defective angiogenesis, oxidative stress, and dicarbonyl stress collectively contribute to progressive vascular damage and delayed tissue regeneration.

Unlike previous studies focusing mainly on the antibacterial activity of OSTI-1872, the present work extends the functional spectrum of this amphibian BBI-type peptide by demonstrating its potential role in endothelial protection under MGO-induced metabolic stress. Among the analogues, OSTI-2337 showed the most favourable protective profile, which was associated with improved physicochemical characteristics, including enhanced charge distribution and hydrophilicity, while preserving the conserved BBI-type structural framework. These findings suggest that rational modulation of peptide properties may provide an effective strategy for improving the biological performance of BBI-derived peptides.

Previous amphibian-derived BBI-type peptides have mainly been investigated for serine protease inhibition and antimicrobial properties [21], whereas their potential roles in vascular protection and diabetes-associated endothelial dysfunction remain largely unexplored. In contrast, OSTI-2337 demonstrated protective activity against MGO-induced endothelial injury, suggesting a previously unrecognised biological application of the BBI scaffold. Unlike many peptide-based wound therapies that primarily focus on a single pathological component, such as bacterial elimination or angiogenesis promotion [22], OSTI-2337 combines antibacterial activity with attenuation of oxidative stress and endothelial dysfunction, which may provide additional therapeutic value in diabetic wound-associated vascular dysfunction.

Recent advances in peptide engineering have shifted therapeutic peptide development from single-functional molecules toward multifunctional designs capable of simultaneously targeting infection, oxidative imbalance, inflammation, and tissue regeneration [23,24]. In the context of diabetic wound healing, infection, excessive oxidative stress, chronic inflammation, and impaired angiogenesis are closely interconnected, highlighting the need for therapeutic strategies with broader biological activities. Several engineered peptide systems, including LL-37-derived multifunctional peptides, have shown potential to regulate multiple aspects of the wound microenvironment. In this study, OSTI-2337 represents a structurally optimised amphibian peptide candidate that combines antibacterial activity with endothelial protection and modulation of redox-associated pathways, suggesting its potential value for future investigation in diabetic wound-associated vascular dysfunction.

Methylglyoxal (MGO) is a highly reactive dicarbonyl compound and a key precursor of advanced glycation end products (AGEs), which accumulate under diabetic conditions and contribute to endothelial dysfunction. Reduced NO bioavailability is considered a hallmark of endothelial dysfunction because NO is indispensable for maintaining vascular tone, inhibiting platelet aggregation, suppressing inflammatory activation, and promoting angiogenesis. MGO exposure markedly reduced NO production in HUVECs, accompanied by decreased VEGF expression and reduced eNOS phosphorylation, indicating severe impairment of endothelial function. These alterations were further reflected by the diminished migratory capacity and impaired tube formation observed following MGO treatment [25]. In this study, MGO treatment significantly reduced NO secretion levels in HUVECs, while inhibiting VEGF expression and eNOS phosphorylation, leading to a significant decrease in angiogenesis and successfully establishing an in vitro model of endothelial dysfunction. Treatment with OSTI-1872 and particularly OSTI-2337 significantly restored NO production together with VEGF expression and eNOS phosphorylation. Because VEGF-mediated activation of eNOS represents one of the principal signalling pathways regulating endothelial repair and angiogenesis, restoration of the VEGF/eNOS axis provides a plausible molecular explanation for the enhanced migration and capillary-like tube formation observed after peptide treatment. Although our results consistently demonstrate activation of the PI3K/AKT/GSK3β/Nrf2 signalling axis following OSTI-2337 treatment, causal relationships remain to be confirmed. Future studies employing pathway-specific inhibitors, siRNA-mediated knockdown, or genetic approaches will be necessary to verify whether activation of this pathway is indispensable for the observed endothelial protection.

Further research revealed that structural optimisation markedly enhanced the biological activity of the parent peptide. Natural amphibian peptides often exhibit favourable biological activities but are limited by suboptimal physicochemical properties. Rational modification of amino acid composition, charge distribution, and secondary structural propensity has therefore become an effective strategy for improving peptide performance [21]. In the present study, optimisation of the primary sequence generated OSTI-2337, which consistently outperformed the parent peptide in restoring endothelial function. Circular dichroism analysis further demonstrated that the optimised peptides exhibited increased α-helical propensity under membrane-mimicking hydrophobic conditions. Amphipathic α-helical structures are generally associated with improved membrane interactions and enhanced cellular uptake [26], which may partially explain the superior biological activity observed for OSTI-2337. Importantly, the optimised peptide retained antibacterial activity while simultaneously exhibiting enhanced endothelial protection, highlighting the feasibility of structural modification for developing multifunctional therapeutic peptides. This multifunctional profile suggests that these peptides may be beneficial in pathological conditions involving both endothelial dysfunction and infection, such as diabetic chronic wounds, where reducing microbial burden and improving vascular repair are both critical for effective healing.

Oxidative stress is considered one of the important molecular mechanisms by which MGO induces vascular injury. Excessive intracellular accumulation of reactive oxygen species (ROS) not only directly damages lipids, proteins, and nucleic acids but also disrupts redox-sensitive signalling pathways, ultimately leading to endothelial dysfunction and impaired angiogenesis [27]. In this study, MGO exposure markedly increased intracellular ROS generation and oxidative DNA damage, as evidenced by elevated ROS fluorescence intensity and increased 8-OHdG accumulation. In contrast, treatment with both OSTI-1872 and particularly OSTI-2337 significantly reduced ROS accumulation and attenuated oxidative DNA damage, indicating that the endothelial protective effects of these peptides are closely associated with restoration of intracellular redox homeostasis.

The maintenance of redox homeostasis largely depends on the activation of endogenous antioxidant defence systems. Among these, nuclear factor erythroid 2-related factor 2 (Nrf2) is recognised as the master regulator of cellular antioxidant responses [28]. Under physiological conditions, Nrf2 is retained in the cytoplasm and undergoes continuous ubiquitin-mediated degradation. Following oxidative stimulation, Nrf2 dissociates from its inhibitory complex and translocates into the nucleus, where it binds to antioxidant response elements (AREs) and promotes the transcription of multiple cytoprotective genes, including HO-1, NQO1, and other phase II detoxifying enzymes [29,30,31,32]. Consistent with this regulatory mechanism, MGO treatment markedly reduced both total Nrf2 expression and its nuclear localisation in HUVECs, accompanied by decreased expression of the downstream antioxidant proteins HO-1 and NQO1. Peptide treatment effectively reversed these alterations, suggesting that restoration of Nrf2 signalling contributes to the enhanced antioxidant capacity observed following OSTI-1872 and OSTI-2337 treatment.

Importantly, the present study further explored the upstream signalling events responsible for Nrf2 regulation. Increasing evidence indicates that activation of the PI3K/AKT pathway represents one of the principal mechanisms promoting Nrf2-mediated antioxidant responses. The PI3K/AKT signalling pathway plays an important role in regulating endothelial function, cell survival, and oxidative stress responses. Activated AKT has been shown to phosphorylate glycogen synthase kinase-3β (GSK3β) at Ser9, thereby suppressing its kinase activity. Inactivation of GSK3β prevents phosphorylation-dependent degradation of Nrf2, facilitating its nuclear accumulation and transcriptional activation of antioxidant genes. Therefore, disruption of the PI3K/AKT/GSK3β signalling cascade has become an important molecular feature of endothelial oxidative injury under diabetic conditions [33,34,35,36].

Previous studies have reported that MGO markedly reduced the phosphorylation ratios of PI3K, AKT, and GSK3β, indicating suppression of the PI3K/AKT pathway and consequent activation of GSK3β through reduced inhibitory phosphorylation [37,38]. In the present study, MGO treatment was associated with decreased phosphorylation of PI3K and AKT, along with increased GSK3β expression, indicating disruption of this signalling pathway. Peptide treatment partially restored p-PI3K and p-AKT levels and reduced GSK3β expression. Given that GSK3β has been reported to negatively regulate Nrf2 stability, the increased GSK3β expression observed in this study may contribute to enhanced Nrf2 degradation and subsequent suppression of antioxidant responses, potentially exacerbating oxidative stress [39,40], these findings suggest a coordinated modulation of the PI3K/AKT/GSK3β/Nrf2 signalling axis. Although the present study provides strong evidence supporting the involvement of the PI3K/AKT/GSK3β/Nrf2 signalling cascade, several limitations should be acknowledged. The current conclusions are primarily based on changes in protein phosphorylation and expression. The observed molecular alterations strongly support activation of this pathway, a direct causal relationship between pathway activation and endothelial protection remains to be further verified in future studies. Nevertheless, the high degree of consistency among the phosphorylation of PI3K, AKT, and GSK3β, the restoration of Nrf2 signalling, the upregulation of HO-1 and NQO1, and the reduction in intracellular oxidative stress provides compelling evidence that coordinated regulation of this signalling network represents an important mechanism underlying the vascular protective effects of OSTI-2337.

In addition to restoring antioxidant signalling, another important finding of the present study is the significant recovery of glyoxalase 1 (GLO1) expression following peptide treatment. Unlike conventional antioxidant strategies that primarily target excessive ROS generation, enhancement of the glyoxalase system directly promotes intracellular detoxification of methylglyoxal, thereby reducing dicarbonyl stress at its metabolic source. Increasing evidence indicates that impaired GLO1 expression or activity is closely associated with intracellular MGO accumulation, accelerated AGE formation, mitochondrial dysfunction, and progressive endothelial injury during diabetes [41,42]. Consequently, restoration of GLO1 has recently emerged as a promising therapeutic strategy for limiting MGO-mediated vascular damage.

In the present study, MGO markedly suppressed GLO1 protein expression in HUVECs, whereas treatment with both OSTI-1872 and OSTI-2337 significantly restored GLO1 expression. Although intracellular MGO concentration and GLO1 enzymatic activity were not directly determined, the observed recovery of GLO1 expression suggests an enhanced cellular capacity for MGO detoxification. Together with the reduced ROS accumulation, restoration of antioxidant signalling, and improved endothelial function observed in this study, these findings support the hypothesis that modulation of the glyoxalase system represents an additional protective mechanism underlying the biological activity of amphibian-derived BBI-type peptides.

Interestingly, recent studies have suggested that the glyoxalase system and cellular antioxidant defence are not independent protective pathways but are functionally interconnected [43,44]. Excessive MGO accumulation promotes oxidative stress through protein glycation and mitochondrial dysfunction, whereas oxidative stress further suppresses GLO1 expression and activity, thereby establishing a vicious cycle that amplifies endothelial injury. Consequently, simultaneous restoration of antioxidant defence and MGO detoxification may provide greater protection than targeting either pathway alone. The coordinated recovery of PI3K/AKT/GSK3β/Nrf2 signalling together with increased GLO1 expression observed in the present study, therefore, suggests that OSTI-2337 exerts endothelial protective effects through complementary regulation of redox homeostasis and dicarbonyl detoxification rather than through a single molecular target.

Another noteworthy characteristic of the present study is that both the parent peptide and its optimised analogue retained antibacterial activity. This finding further expands the potential therapeutic application of amphibian-derived BBI-type peptides. Diabetic chronic wounds are characterised not only by persistent endothelial dysfunction and impaired angiogenesis but also by recurrent bacterial infection, prolonged inflammation, and delayed tissue regeneration. Persistent bacterial colonisation can further aggravate oxidative stress and inflammatory responses, thereby creating an unfavourable microenvironment for vascular repair and wound healing. Therefore, therapeutic strategies that simultaneously reduce bacterial burden and improve endothelial function may provide additional advantages for diabetic wound management. Although the antibacterial activity of OSTI-2337 was evaluated only under in vitro conditions in the present study, its activity against several wound-associated bacterial strains, together with its protective effects against MGO-induced endothelial injury, suggests that OSTI-2337 may represent a multifunctional peptide candidate with potential relevance to both microbial infection and vascular dysfunction. Further investigations using infected diabetic wound models are required to determine its in vivo therapeutic efficacy.

Although the present study focused primarily on the biological effects of OSTI-2337, its future therapeutic development will also depend on favourable stability and pharmacological properties. The disulfide-rich BBI-type scaffold may provide intrinsic structural stability and improved resistance to proteolytic degradation. Previous investigations [21] of related OSTI-derived peptides have suggested favourable stability profiles; however, the pharmacokinetics, biodistribution, immunogenicity, and in vivo efficacy of OSTI-2337 remain to be determined in future studies. These aspects, together with further validation of its biological mechanisms and therapeutic effects, represent important considerations for the future development of OSTI-2337.

Several limitations of the present study should also be acknowledged. First, although HUVECs provide a well-established model for studying endothelial dysfunction, all cellular experiments were performed using an in vitro MGO-induced HUVEC injury model. Therefore, further validation using additional endothelial cell models and in vivo diabetic wound models will be necessary to confirm the translational potential of OSTI-2337. Second, although the PI3K/AKT/GSK3β/Nrf2 signalling pathway was significantly restored following peptide treatment, pharmacological inhibition or genetic knockdown experiments were not performed to establish a direct causal relationship between pathway activation and endothelial protection. Future studies using pharmacological inhibitors, siRNA-mediated knockdown, or other genetic strategies will be required to determine whether this signalling axis is necessary for the protective effects of OSTI-2337. Third, only GLO1 protein expression was evaluated, whereas GLO1 enzymatic activity, intracellular MGO concentration, AGE accumulation, and other components of the glyoxalase system were not determined. These issues warrant further investigation to clarify the precise molecular mechanisms responsible for peptide-mediated vascular protection.

In conclusion, the present study demonstrates that structural optimisation of the amphibian-derived BBI-type peptide OSTI-1872 generated a new analogue, OSTI-2337, with enhanced endothelial protective activity. The significance of this work lies not only in the observation that OSTI-2337 reduces oxidative stress, but also in the mechanistic connection between peptide structural optimisation, endothelial repair, PI3K/AKT/GSK3β/Nrf2-mediated antioxidant defence, and GLO1-associated MGO detoxification. This coordinated regulation of redox homeostasis and the glyoxalase system distinguishes OSTI-2337 from a simple antioxidant candidate and supports its potential as a multifunctional peptide lead for diabetes-associated vascular injury and chronic wound-related complications.

## 5. Conclusions

In summary, this study provides evidence that amphibian-derived BBI-type peptides possess protective potential against MGO-induced endothelial dysfunction, expanding the known biological functions of this peptide family. Structural optimisation led to the identification of the analogue OSTI-2337, which exhibited superior endothelial protective activity while retaining antibacterial properties. These findings demonstrate that rational peptide engineering can enhance the functional potential of amphibian-derived BBI-type peptides and support OSTI-2337 as a promising candidate for further investigation in diabetes-associated vascular injury and chronic wound-related complications. Further studies using in vivo models and pathway-specific validation approaches will be required to clarify its therapeutic potential.

## Figures and Tables

**Table 1:** Peptide sequences and physicochemical properties.

Peptides	Sequences	Hydrophilicity	Net Charge
OSTI-1872	AALKGCWTKSIPPRPCF-NH_2_	−0.21	3.91
OSTI-1853	AALKGCWTKSIPPRPC**K**-NH_2_	0.11	4.91
OSTI-1840	AALKGCWTKS**KPPK**PCK-NH_2_	0.39	5.91
OSTI-2337	**ALKRALKR**CWTKSKPPKPCK-NH_2_	0.70	8.91

Note: The hydrophilicity and theoretical net charge values were calculated using the BACHEM Peptide Calculator at pH 7.0. Bolded residues indicate amino acid substitutions or newly introduced sequences compared with the parent peptide OSTI-1872, whereas underlined sequences represent the highly conserved BBI-type loop region.

**Table 2:** The MIC/MBC values (μM) of peptides against eight microorganisms.

Bacteria Strains		OSTI-1872	OSTI-1853	OSTI-1840	OSTI-2337
Gram-negative bacteria	*E. coli* (ATCC CRM 8739)	128/256	64/64	16/16	16/16
	*P. aeruginosa* (ATCC CRM 9027)	256/512	128/128	128/128	64/64
	*K. pneumoniae* (ATCC CRM 43861)	>512	128/128	32/32	128/128
	*A. baumannii* (BAA 747)	>512	>512	>512	512/512
Gram-positive bacteria	*S. aureus* (ATCC CRM 6538)	>512	>512	>512	>512
	*E. faecium* (NCTC 12697)	>512	>512	>512	>512
	MRSA (NCTC 12493)	>512	128/128	256/512	64/64
Fungus	*C. albicans* (ATCC 10231)	>512	>512	>512	>512

## Data Availability

The data supporting the findings of this study are available within the article and its Supplementary Information Files.

## Supplementary Materials

The following supporting information can be downloaded at: https://www.mdpi.com/article/10.3390/biom16081157/s1, Supplementary Information Files: The raw data of Western blots.
