# Supplementary material for: Structural Optimisation of an Amphibian BBI-Type Peptide Enhances Endothelial Protection Against Methylglyoxal-Induced Injury Through Coordinated Regulation of Glyoxalase-Mediated Detoxification and Redox Homeostasis
Source: Biomolecules. 2026 Aug 9;16(8):1157. doi: 10.3390/biom16081157 (PMC13511522; doi:10.3390/biom16081157)
Supplement: Supplementary file 1 [file biomolecules-16-01157-s001.zip › biomolecules-4466383-supplementary.pdf]

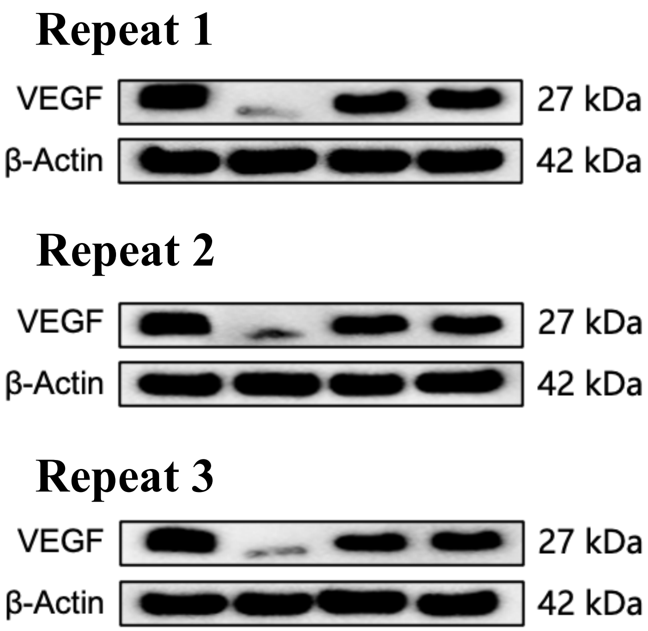
Repeat 1
VEGF
27 kDa
β-Actin
42 kDa
Repeat 2
VEGF
27 kDa
β-Actin
42 kDa
Repeat 3
VEGF
27 kDa
β-Actin
42 kDa

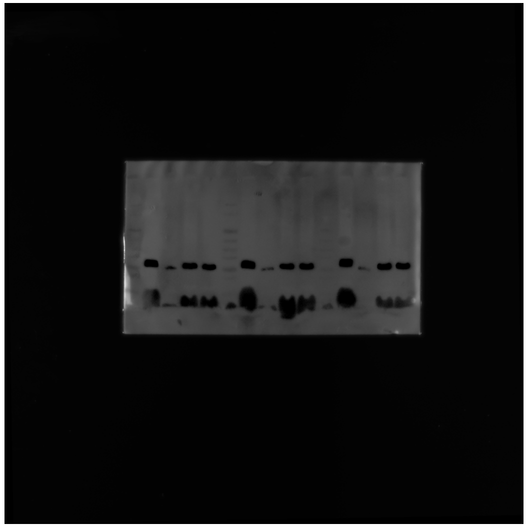

VEGF

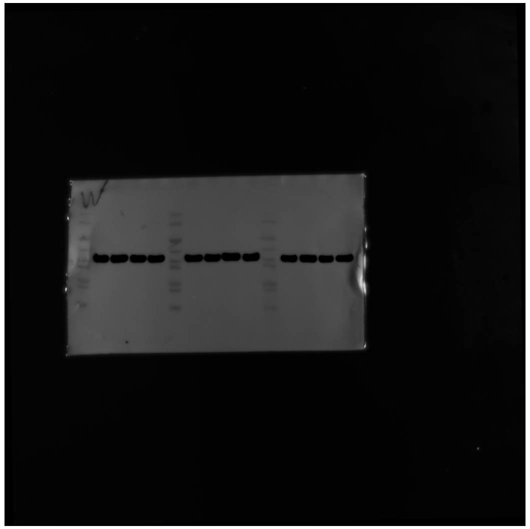

β-actin

Repeat 1

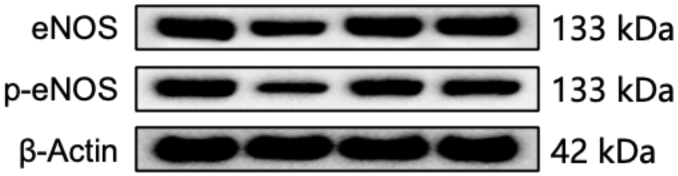

Repeat 2

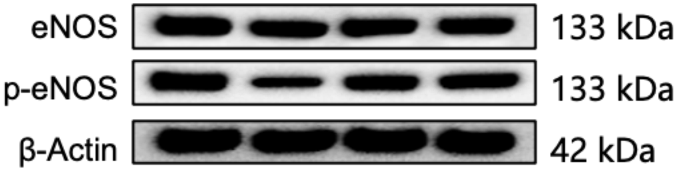

Repeat 3

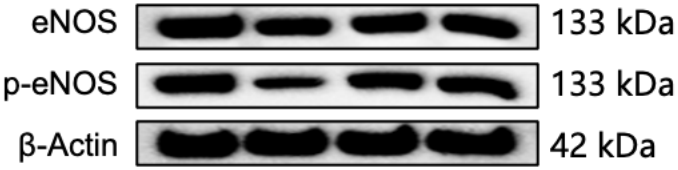

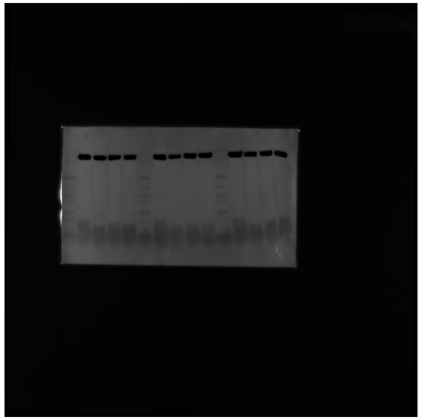

eNOS

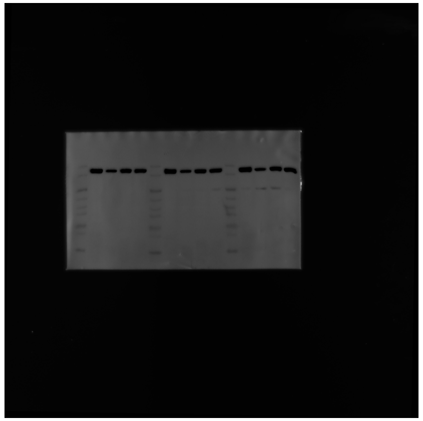

p-eNOS

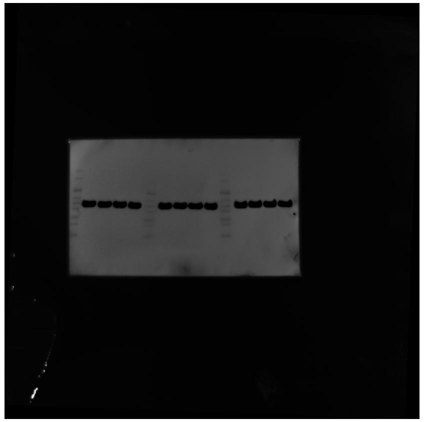

β-actin

Repeat 1

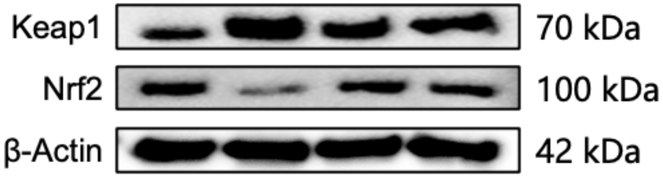

Repeat 2

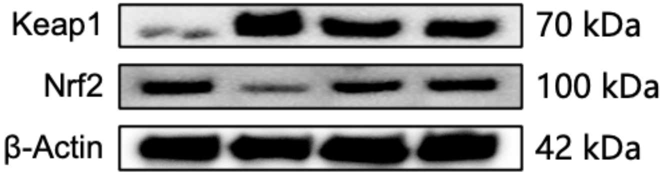

Repeat 3

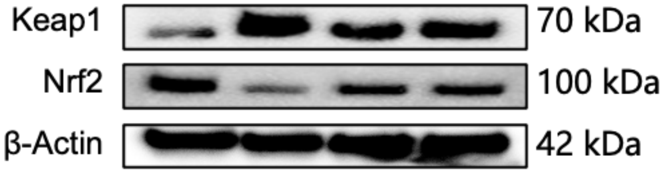

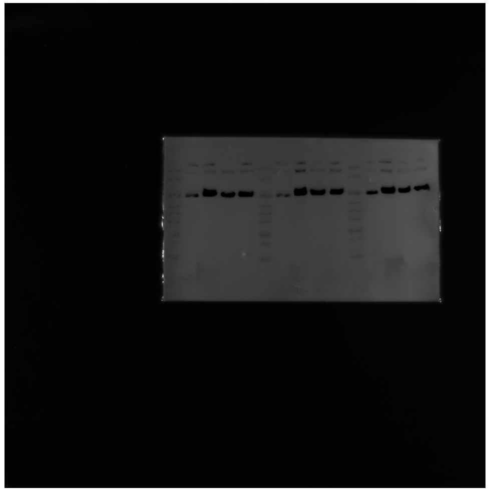

Keap1

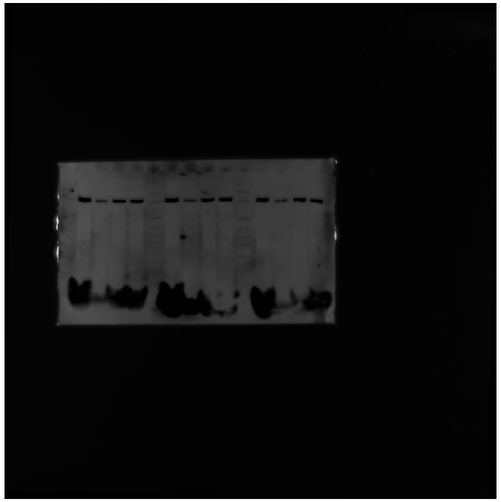

Nrf2

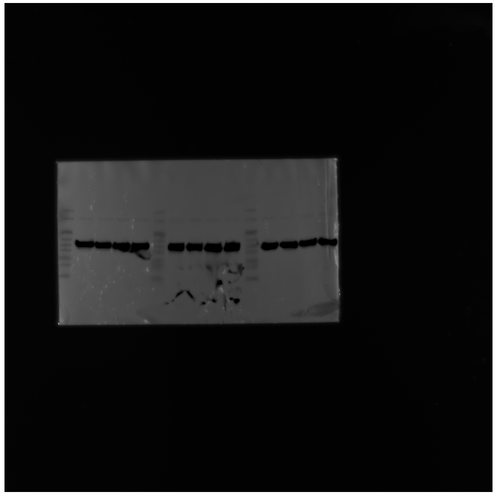

β-actin

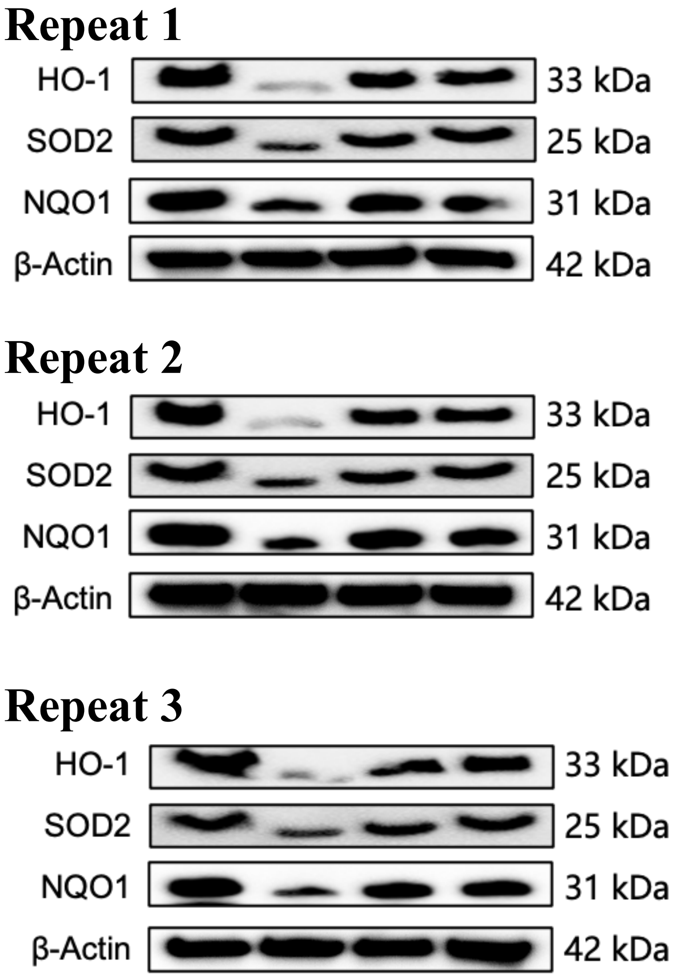

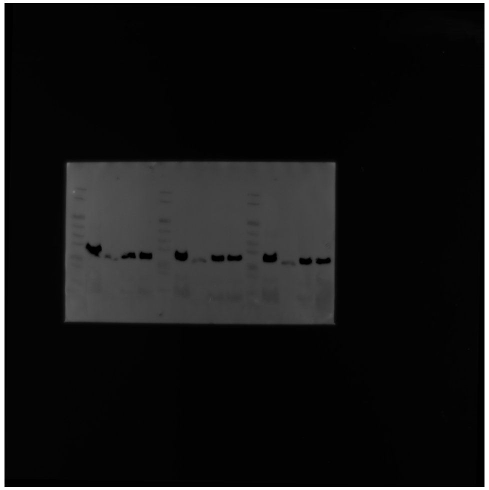

HO-1

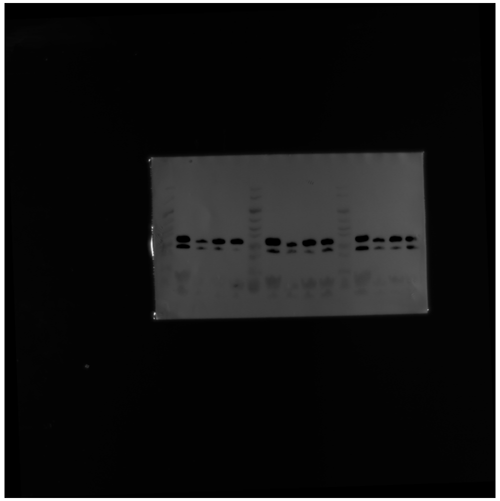

NQO1

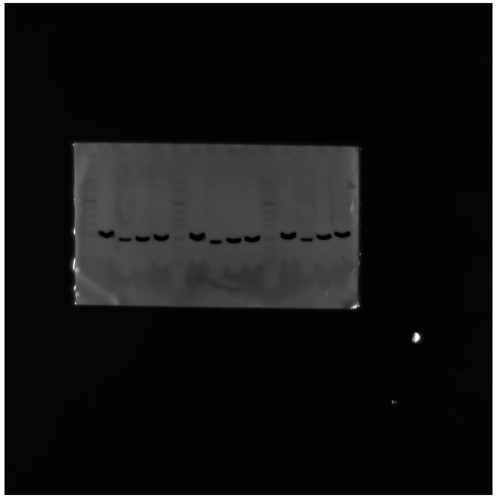

SOD2

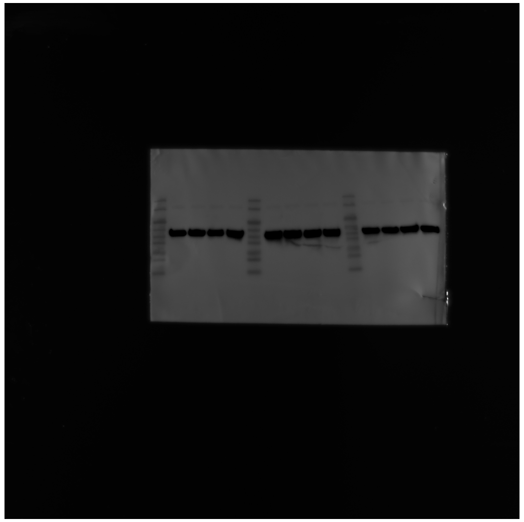

β-actin

Repeat 1

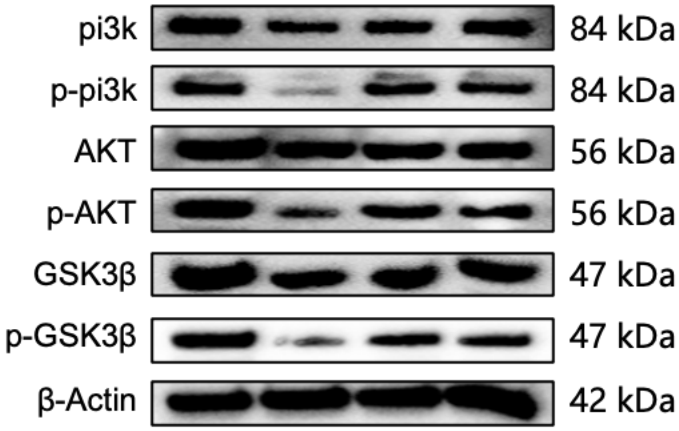

Repeat 2

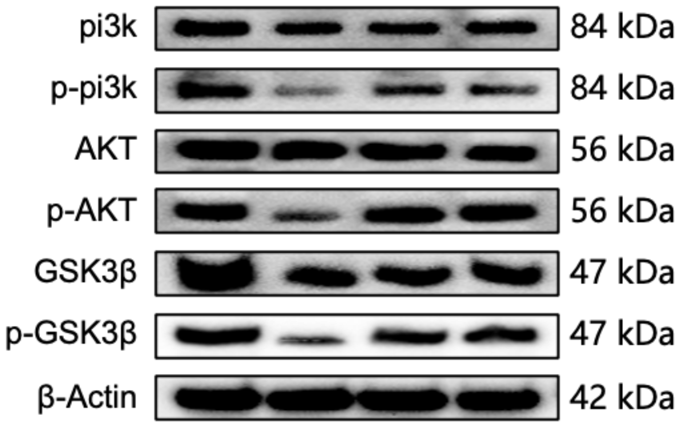

Repeat 3

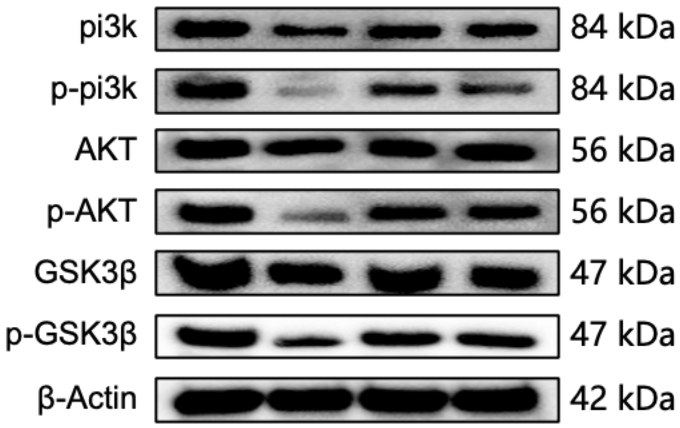

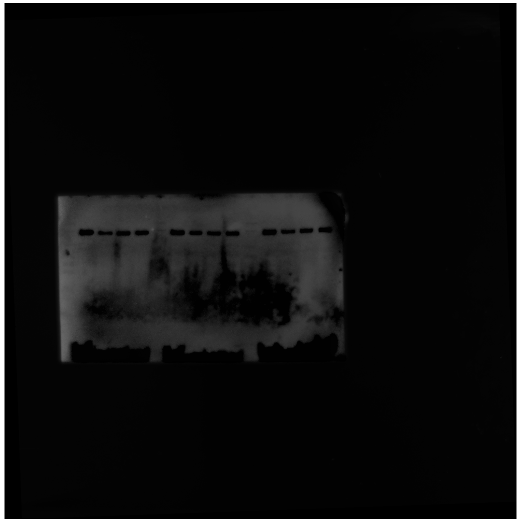

pI3K

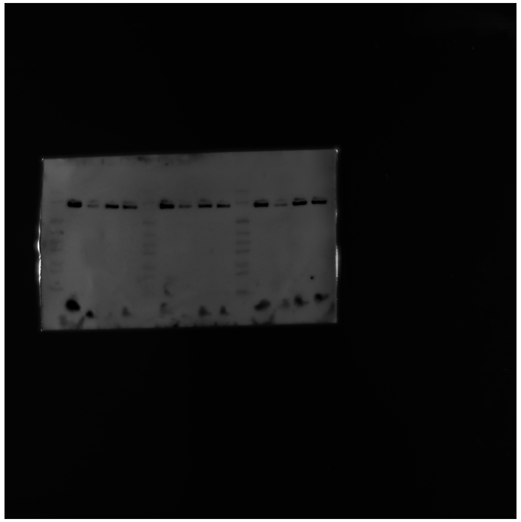

p-pI3K

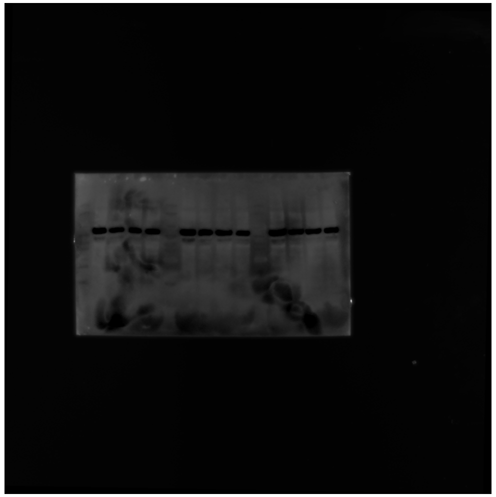

AKT

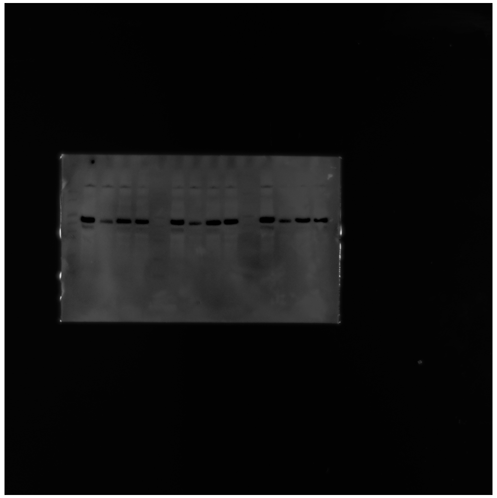

p-AKT

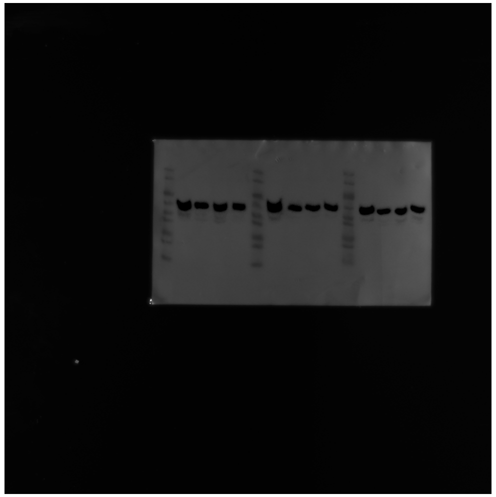

GSK3β

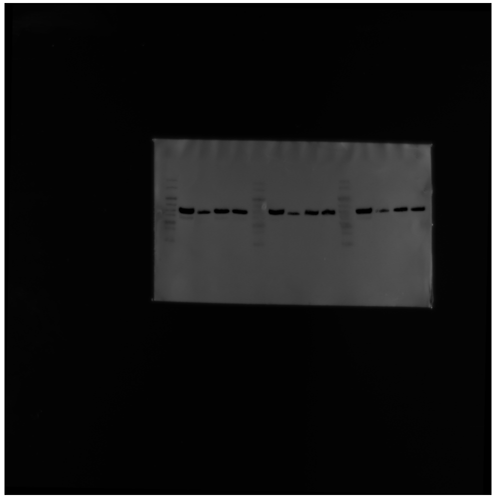

p-GSK3β

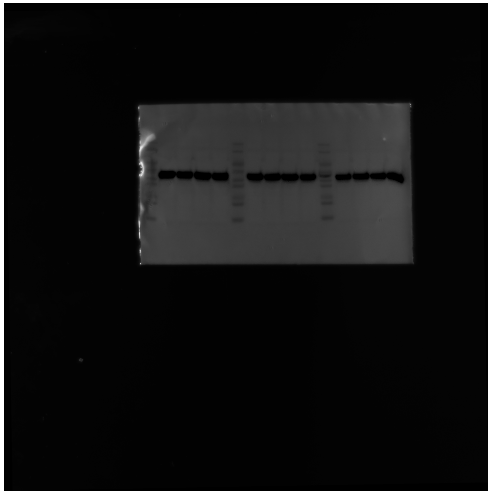

β-actin

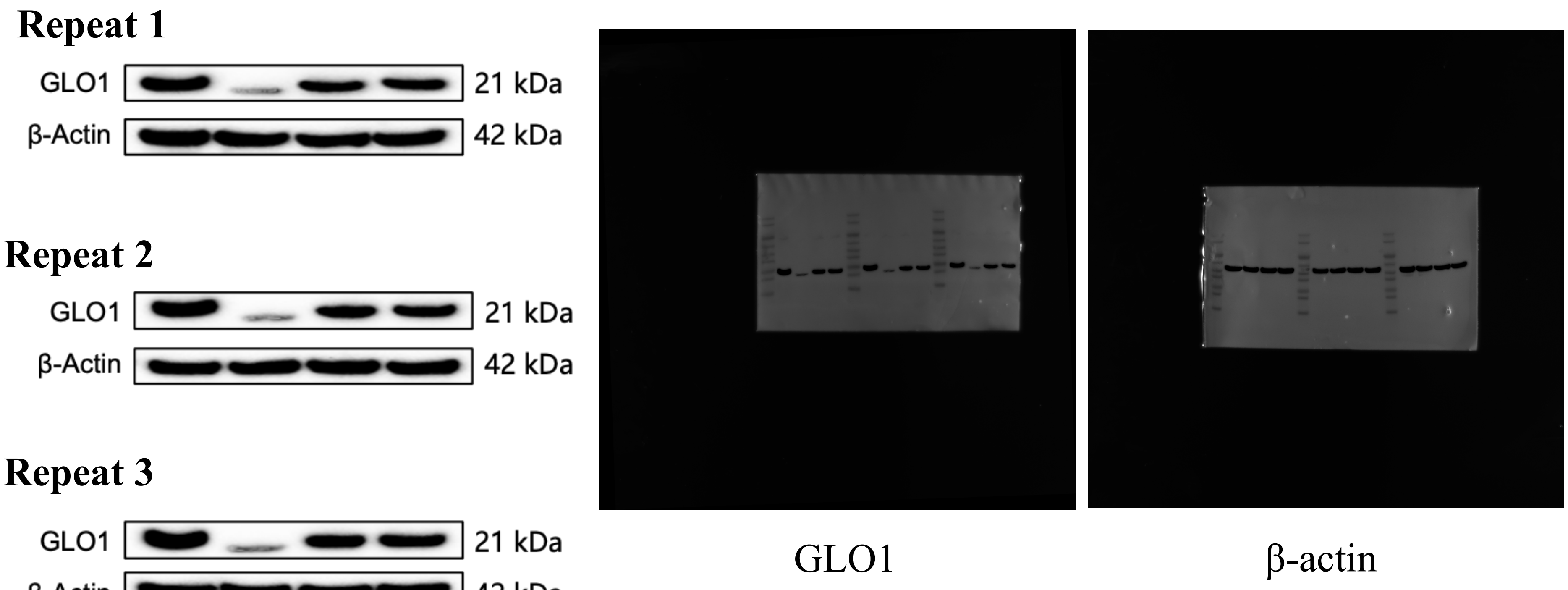
Repeat 1
GLO1
21 kDa
β-Actin
42 kDa
Repeat 2
GLO1
21 kDa
β-Actin
42 kDa
Repeat 3
GLO1
21 kDa
β-Actin
42 kDa
GLO1
β-actin

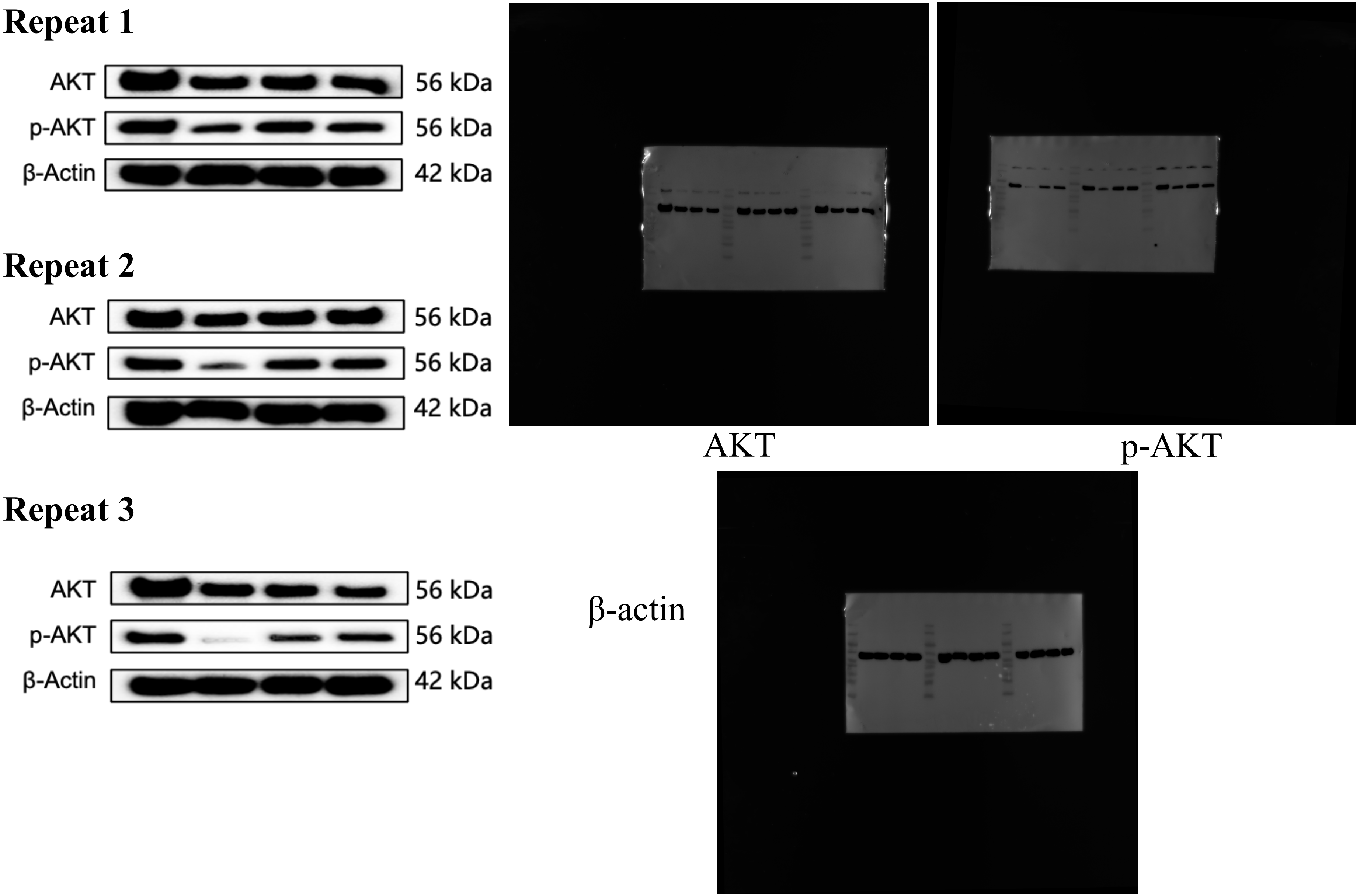
Repeat 1
AKT
56 kDa
p-AKT
56 kDa
β-Actin
42 kDa
Repeat 2
AKT
56 kDa
p-AKT
56 kDa
β-Actin
42 kDa
Repeat 3
AKT
56 kDa
p-AKT
56 kDa
β-Actin
42 kDa
AKT
p-AKT
β-actin
